# Assessment of phenotypic trait plasticity in the oilseed *Camelina sativa* using integrated early stage abiotic stress and field studies

**DOI:** 10.1093/plphys/kiag052

**Published:** 2026-02-11

**Authors:** Susana Silvestre, Sylvain Prigent, Pierre Pétriacq, Kirsty L Hassall, Malo Le Boulch, Anais Da Costa, Frédérique Tellier, Barbara Alberghini, Emilio Aldorino, Asis Hallab, Dominik K Großkinsky, Cédric Cassan, Andrea Monti, Javier Prieto, Paloma Leon, Yuri Herreras Yambanis, Yves Gibon, Björn Usadel, Jean-Denis Faure, Federica Zanetti, Claudia Jonak, Richard P Haslam

**Affiliations:** Rothamsted Research, Harpenden AL5 2JQ, United Kingdom; University of Bordeaux, INRAE, UMR1332 BFP, Villenave d’Ornon 33882, France; Bordeaux Metabolome, MetaboHUB, PHENOME-EMPHASIS, Villenave d’Ornon 33140, France; University of Bordeaux, INRAE, UMR1332 BFP, Villenave d’Ornon 33882, France; Bordeaux Metabolome, MetaboHUB, PHENOME-EMPHASIS, Villenave d’Ornon 33140, France; Rothamsted Research, Harpenden AL5 2JQ, United Kingdom; Department of Statistics, University of Warwick, Coventry CV4 7AL, United Kingdom; University of Bordeaux, INRAE, UMR1332 BFP, Villenave d’Ornon 33882, France; Université Paris-Saclay, INRAE, AgroParisTech, Institut Jean-Pierre Bourgin for Plant Sciences (IJPB), Versailles 78000, France; Université Paris-Saclay, INRAE, AgroParisTech, Institut Jean-Pierre Bourgin for Plant Sciences (IJPB), Versailles 78000, France; Department of Agricultural and Food Sciences (DISTAL), Alma Mater Studiorum—Università di Bologna, Bologna, Italy; Rothamsted Research, Harpenden AL5 2JQ, United Kingdom; IBG-4 Bioinformatics, CEPLAS, Forschungszentrum Jülich, Wilhelm Johnen Str, Jülich 52428, Germany; Applied Bioinformatics, University of Applied Sciences Bingen, Berlinstraße 109, Bingen on the Rhine 55411, Germany; Centre for Health & Bioresources, Bioresources Unit, AIT Austrian Institute of Technology, Tulln 3430, Austria; University of Bordeaux, INRAE, UMR1332 BFP, Villenave d’Ornon 33882, France; Bordeaux Metabolome, MetaboHUB, PHENOME-EMPHASIS, Villenave d’Ornon 33140, France; Department of Agricultural and Food Sciences (DISTAL), Alma Mater Studiorum—Università di Bologna, Bologna, Italy; Camelina Company, Camino de la Carrera, 11-11, Fuente el Saz de Jarama, Madrid 28140, Spain; Camelina Company, Camino de la Carrera, 11-11, Fuente el Saz de Jarama, Madrid 28140, Spain; Camelina Company, Camino de la Carrera, 11-11, Fuente el Saz de Jarama, Madrid 28140, Spain; University of Bordeaux, INRAE, UMR1332 BFP, Villenave d’Ornon 33882, France; Bordeaux Metabolome, MetaboHUB, PHENOME-EMPHASIS, Villenave d’Ornon 33140, France; IBG-4 Bioinformatics, CEPLAS, Forschungszentrum Jülich, Wilhelm Johnen Str, Jülich 52428, Germany; Faculty of Mathematics and Natural Sciences, Institute for Biological Data Science, Heinrich Heine University, Düsseldorf 40225, Germany; Université Paris-Saclay, INRAE, AgroParisTech, Institut Jean-Pierre Bourgin for Plant Sciences (IJPB), Versailles 78000, France; Department of Agricultural and Food Sciences (DISTAL), Alma Mater Studiorum—Università di Bologna, Bologna, Italy; Centre for Health & Bioresources, Bioresources Unit, AIT Austrian Institute of Technology, Tulln 3430, Austria; Rothamsted Research, Harpenden AL5 2JQ, United Kingdom

## Abstract

Connecting the characterization of juvenile (pre-anthesis) plant stress responses in controlled environments to field agronomic performance is a challenge. The oilseed crop *Camelina sativa* (camelina), with its innate resilience and plasticity, presents an opportunity to understand the underlying mechanisms of juvenile resilience and identify the implications for yield in diverse pedoclimates. A better understanding of camelina's abiotic stress resilience is important in the context of climate change and the development of breeding programs for climate-tolerant crops. In this study, 54 accessions representing the genetic diversity observed in the wider publicly available population were used to investigate the plasticity of camelina's early stage response to drought and heat stress, combined with an evaluation of field performance in multilocation field trials. A combinatorial phenotyping approach of early stage drought and heat stress identified stress-responsive signatures within the diversity panel. The substantial variation in the morphophysiological line-specific responses to stress indicated that juvenile and mature camelina plants have significant plasticity and access different stress response strategies. In response to stress, we observed significant molecular metabolic adjustment alongside significant lipid remodeling and physiological compensation. Camelina was resilient to drought stress, and certain metabolites were identified as indicators of abiotic stress response. Applying an integrated approach, early stage phenotyping and multilocation field trials provided a complete assessment of the camelina stress response and facilitated a connection to crop productivity. This approach facilitates improved breeding programs, addresses the restrictions of limited genetic diversity in camelina, and supports the development of local varieties optimized for climate resilience.

## Introduction

Agriculture faces increasing threats from extreme weather events associated with anthropogenic climate change (Ray et al 2015; Lesk et al 2016). Fluctuating hot and dry conditions associated with elevated temperatures and irregular precipitation poses a particular risk to major staple crops (Lesk et al 2021) and often result in significant yield reduction. Heat and drought stress restrict germination and impair development, growth, and reproduction. Temperate Brassica oilseeds, like oilseed rape (*Brassica napus*), are particularly sensitive to increasing temperatures and water limitation (Secchi et al 2023). Research has established a negative relationship between abiotic (heat and drought) stress and agronomic performance, eg seed yield and quality (Jensen et al 1996; Yu et al 2014), although the impact of abiotic stress on seed oil biosynthesis and regulation is still uncertain.

Diversifying cropping systems with better-adapted oilseeds could potentially stabilize productivity under changing environments associated with climate change. Growers are returning to traditional oilseeds like *Camelina sativa* L. Crantz (camelina), a *Brassicaceae* crop with a recognized commercial value that results from its seed oil quality, ie elevated levels of omega-3 α-linolenic acid, which is nutritionally important. Beyond nutrition, camelina seed oil is widely used, eg food, biofuel, and feed stock for biobased industries. Moreover, camelina has resilience to challenging environments and can be utilized in different cropping systems. Its 2 crop biotypes, spring and winter (Zubr 1997), and suitability for different pedoclimates further expand its cultivation area and utilization. Furthermore, the low agricultural input requirement (fertilization) and short growing cycle make camelina a good candidate for expansion into marginal land not commercially cultivated (Zanetti et al 2021). As an ancestral crop, camelina genetic diversity is only now receiving attention (Luo et al 2019; Decker et al 2025). Studies have focused on genomic and seed traits among natural accessions and breeding lines to understand the genetic mechanisms underlying productivity (Vollmann et al 2007; King et al 2019; Hotton et al 2020; Li et al 2021). Recognized for its tolerance to biotic and abiotic stresses, camelina is a good model to study plasticity (Großkinsky et al 2023). In comparison with oilseed rape, Gao et al (2018) reported that camelina showed more tolerance to drought stress. However, the ability of camelina to tolerate extreme drought stress varies with genotype (Čanak et al 2020) indicating untapped potential. Camelina accessions and cultivars grow in varying climatic niches throughout the globe, and temperature was found to elicit plasticity in camelina seed oil (Brock et al 2020). The impact of postanthesis heat stress on oil yield and fatty acid (FA) composition in camelina has been established, reducing seed yield, weight, and oil content and altering FA composition (Nadakuduti et al 2023; Smith and Lu 2024). Several approaches, such as quantitative trait locus (QTL) mapping, genome-wide association, and comparative transcriptome profiling have also begun to characterize the pathways controlling heat tolerance during reproduction (Smith et al 2024). In the field, abiotic stress can affect plants at any point during development. In young tissues under abiotic stress, there is evidence for higher plasticity in traits associated with growth responses (reviewed in Rankenberg et al 2021). However, experimental evidence and understanding early stage abiotic stress responses are limited. Despite the recognized abiotic stress tolerance and plasticity of camelina, no studies have investigated how it responds to heat and drought in early growth stages. To address this complexity, metabolomic and biochemical profiling can now tell us about the biology of the plant response to abiotic stress and support genomic selection, by identifying traits of interest (Alwood et al 2021; Fernandez et al 2021).

Effective solutions to develop stress-tolerant crops require the inclusion of realistic agricultural field studies in fundamental research (Prado et al 2025). To address this challenge, we investigated the physiological and molecular mechanisms underlying stress resilience using a combinatorial approach in multiple settings. Improving tolerance to elevated temperatures and drought is essential for camelina agronomic sustainability in growing seasons with increasing meteorological instability. The objectives of this study were to increase our understanding of the camelina multilevel abiotic stress response, characterize the plasticity of this response in different lines, and establish how the diversity of stress responses translates into improved agronomic performance in the field. To achieve this, a combinatorial approach was used to monitor dynamic changes in morphological, physiological, and metabolic responses of a camelina diversity collection to early stage heat and drought stress. In parallel, the collection was grown in multilocation field trials (the United Kingdom, Italy, and France) with diverse environments providing real-world heat and drought conditions, enabling a fully integrated evaluation of the stress response in camelina, plasticity, and connection to agronomic performance. These results offer diagnostic markers and a framework to assess crop stress resilience, advancing the development of climate-resilient oilseeds and facilitating the integration of genetic diversity into cropping systems.

## Materials and methods

### Plant material

The 54 camelina lines were assembled from public collections (Table S1). The panel was selected based on specific distinguishing features, namely genetic diversity, growth cycle length, performance under different climatic conditions, yield, and lipid profile. Prior to glasshouse experimentation and distribution for field trials, the panel was propagated in field trials of replicated plots at INRAe Versailles, France (Spring/Summer 2020).

### Diversity panel population structure analysis

Leaf DNA was extracted using the Nucleospin Kit (Machery-Nagel, Düren, Germany) according to manufacturer. DNA was sequenced using paired end short read sequencing and analyzed alongside whole genome resequencing data of publicly available camelina accessions (Luo et al 2019; Li et al 2021). The resulting data were trimmed with trimmomatic (Bolger et al 2014) and mapped to camelina reference genome (PRJA264159). Alignment of sequencing reads was done with Burrows-Wheeler Aligner software (version 0.7.17). Genomic variants were identified through the characterization of single-nucleotide polymorphisms (SNPs) (GATK software tool version 0.7.44.1.4.0; Poplin et al 2018). PLINK (Purcell et al 2007) was used to carry out the principal component analysis (PCA) of camelina genetic diversity. ADMIXTURE was used to infer each accession genome's fractional composition (Alexander et al 2009), represented by bar plots where each accession was represented by a single bar subdivided into colored sections. Within ADMIXTURE, a cross-validation assay to infer the error bias imposed by assuming the respective number of ancestral populations was performed. By comparison of this cross-validation error with runs using different numbers of ancestral populations, the best fitting number of populations is then found as the one producing the lowest cross-validation error (CVE). Eight populations were found to be optimal with a CVE of 0.47. Each section represents the fraction of a genome coming from ancestry belonging to the subpopulation represented by that color. All code and plots used to carry out these analyses and a detailed description of the procedures are hosted on GitHub (https://github.com/usadellab/untwist). These files are also stored in the supplementary archive “Untwist_Population_Structure_Analysis.zip.”

### Trait measurements in early stage drought and heat experiments

Three treatments were tested in the glasshouse: ambient temperature (20/18 °C day/night) and well-watered (50% soil water content, SWC); ambient temperature (20/18 °C day/night) and drought; and high temperature (32/25 °C day/night) under well-watered conditions. SWC (each individual pot weighed) and BBCH scale (Biologische Bundesantalt, Bundessortenamt and Chemische Industrie; Martinelli and Galasso 2011) were monitored every day in all pots. Leaf length/width (cm) and leaf chlorophyll index were measured using a portable self-calibrating chlorophyll meter SPAD (Chlorophyll Meter SPAD-502 Plus, Konica Minolta Optics, Inc.), monitored every other day in all pots. Ambient and heat stress pots were kept at approx. 50% SWC. Drought was imposed by water withdrawal until each pot reached 15% to 20% SWC, which was maintained thereafter (Fig. S1a). Main stem width was measured with a calliper at the base of the shoot, and BBCH scale was derived from leaf number. BBCH and leaf length were used to monitor growth and stress impact, as it was considered that morphological changes (eg, slower growth rate) were an indicator of stress. Leaf samples were collected for further analysis as detailed in Supplementary Methods.

### Metabolomic analysis

Targeted and untargeted metabolomic techniques were used to cover primary and secondary leaf metabolites (Luna et al 2020; Dussarrat et al 2022). Robotized high-throughput ethanol extraction was used to obtain semipolar metabolites from 20 mg of freshly frozen ground leaves (Luna et al 2020). Quantitative profiling of major compounds of central metabolism included starch, sucrose, glucose, total proteins, total amino acids, malate, citrate, total polyphenols, and chlorophylls *a* and *b*, using targeted assays (Poucet et al 2021). Methods for untargeted metabolic profiling and analysis are detailed in Supplementary Methods.

### Total antioxidant capacity

Total antioxidant capacity (TAC) was colorimetrically determined in a 96-well microplate format by using a commercial kit (Abbexa Ltd., Cambridge, UK; (Stasnik et al 2022). Results were normalized as units (U) per mg protein in leaf extracts. Quadruplicate aliquots were used to quantify protein content using commercial ROTIQuant Bradford solution (Carl Roth GmbH + Co. KG, Karlsruhe, Germany) according to the supplier's instructions.

### Fatty Acid analysis

Field grown seed fatty acid (FA) analysis was performed at INRAe AgroParisTech, France, and at Rothamsted Research (RRes), United Kingdom. Leaf FA analysis of the glasshouse trials was performed at RRes. Both labs used FA methylation protocols analyzed by gas chromatography (Supplementary Methods). A reference camelina line was analyzed by both labs, from which a linear equation was derived using the quantified amounts (µg/mg) of each FA. The linear equation was applied as a correction factor to the datasets, from which FA total content and relative composition were calculated.

### Stable isotope determination

Carbon (C) and nitrogen (N) content and their isotopic ratios were measured by an elemental analyzer (Flash 2000, Thermo Fisher Scientific) coupled with an isotopic ratio mass spectrometer (Delta V Advantage, Thermo Fisher Scientific). Additional details are provided in the Supplementary  Methods.

### Camelina diversity panel multilocation field trials

Field trials were conducted in 3 locations across Europe: Bologna (Italy), Versailles (France), and Harpenden (United Kingdom). In Italy, the diversity panel was sown on 29 November 2020 using a precision plot-drill at a rate of 500 seeds/m^2^. The experimental layout was a completely randomized block with 4 replicates. In France, the diversity panel was sown on 26 March 2021 with a Wintersteiger Rowseed 1R single row seeder at a rate of 500 seeds/m^2^. In both locations the experimental layout was a completely randomized block with 4 replicates. In the United Kingdom, the diversity panel was sown on 19 April 2021 by shallow drilling the camelina seeds with a Haldrup S-25 drill, which followed a nonresolvable block design with 4 blocks of 56 plots each allowing for a total of up to 5 plot replicates per line. Further details, and a summary of the main dates and meteorological data by location can be found in Supplementary  Methods and Table S7. Harvest was done at full maturity, and all yield values were reported on a dry matter basis. Subsamples of seed from each plot were collected and cleaned for further analysis.

### Crop cycle and meteorological data

Location, sowing date, harvest date, and meteorological data of the trials can be found in Supplementary Methods. Temperature (T) and precipitation data were collected by a weather station located onsite. Growing degree days (GDD) were calculated as GDD = Σ[(T_max_ − T_min_)/2 − T_base_]; base temperature used was 4 °C (Gesch and Cermak 2011).

### Seed oil content

Seed oil content was measured by low-resolution time domain Nuclear Magnetic Resonance spectroscopy using a Minispec MQ20 (Bruker) fitted with a robotic sample-handling system (Rohasys). The oil and moisture calibration were constructed according to the manufacturer's instructions using 9 approximately 0.5 g oilseed seed samples ranging between 5% and 10% moisture content and between 30% and 55% oil content (*r*^2^ > 0.99). Camelina seed with known oil and moisture content were supplied by Camelina Company España (Spain) for method verification. Approximately 1 g of seed was used; seeds were kept in the room alongside the instrument for 24 hours prior to analysis to ensure equilibration with room temperature and humidity.

### Thousand grain weight analysis

In Italy, thousand grain weight (TGW) was determined using the Seed Counter S-25 machine by Data Technologies (DATA Detection Technologies Ltd., IL) at the Seed Research and Testing Laboratory (LaRAS) of the University of Bologna. In the United Kingdom, TGW was determined using the MARVIN Digital Seed Analyser (MARViTECH GmbHGermany) to count the number of seeds in approximately 1 g seed sample. In France, TGW was determined using the elmor C3 High Sensitive Seed Counter (Elmor, Switzerland).

### Statistical analysis

The early stage drought and heat stress experiment layout was a resolvable block design spreading the 54 panel lines over 6 blocks (trays), 9 pots per tray. This was replicated 5 times and 3 independent randomizations done, one for each stress. Linear mixed models were fitted using restricted maximum likelihood to each variable with random structure accounting for the blocking imposed in the design (compartment/tray/pot) and treatment structure Line * Stress. Approximate *F* tests were calculated using the Satterthwaite approximation. All models were fitted in R using the lme4 and lmerTest packages. PCA of the effect of stress on measured traits, biomass components, and parameters from the camelina diversity panel glasshouse early stage study were implemented in R v.4.1 (R Core Team 2018) using BioStatFlow tools (v2.9). Multivariate analyses (PCA, Volcano plots) of metabolomic data were performed using MetaboAnalyst v5.0 (Pang et al 2021) with normalized data (median normalization, cube root transformation, and Pareto scaling).

## Results

### Genetic diversity and population structure in the camelina study panel

**Figure 1:**
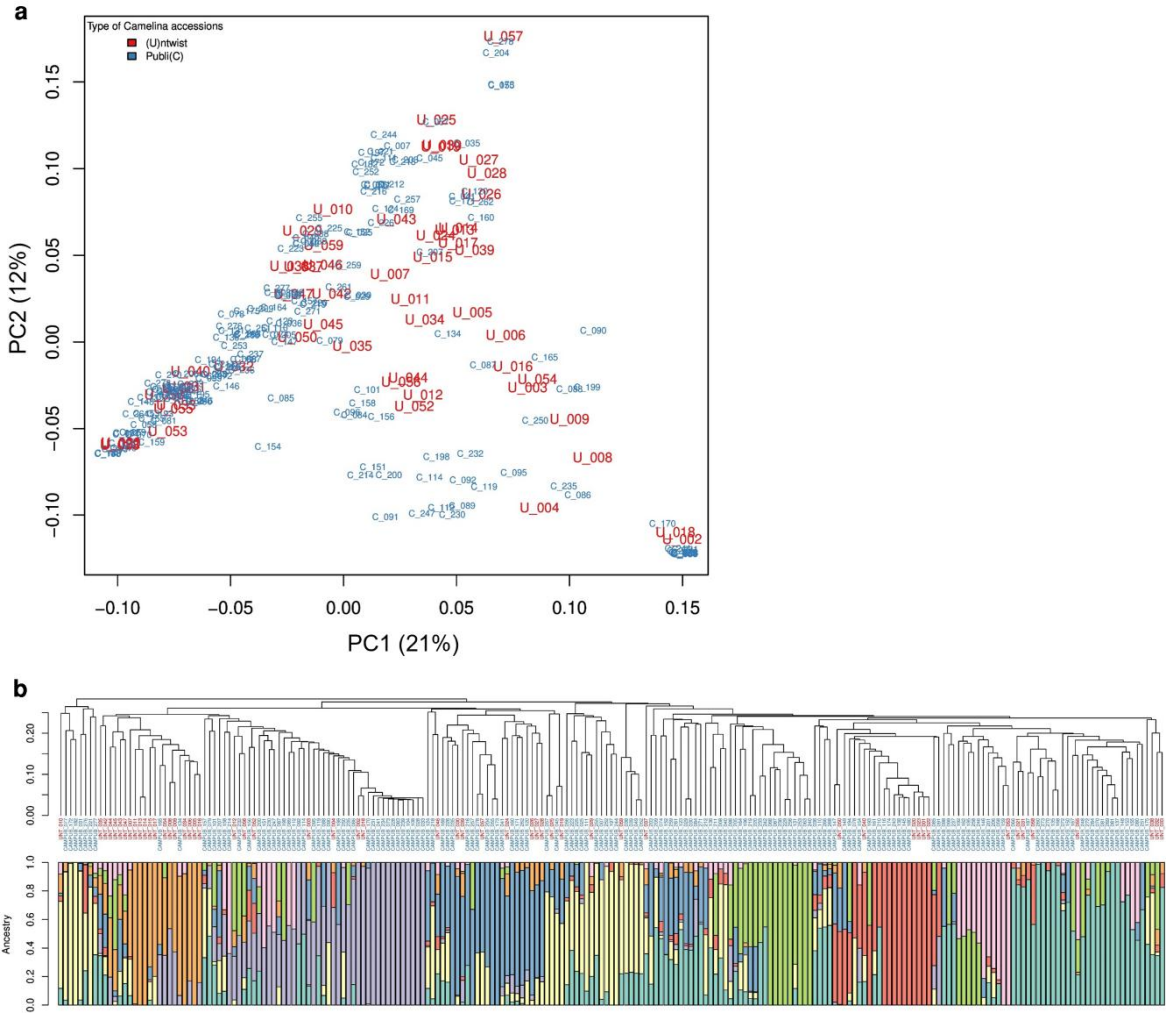
Genetic diversity in the study panel and publicly available camelina lines. a) Scatterplot of the principal component analysis (PCA) of publicly available (blue) and the study panel (red) camelina lines. Lines were abbreviated with a “C_” (public) or a “U_” (study panel) prefix followed by their accession number. b) Admixture population genetic analysis (bottom) aligned with the hierarchical clustering dendrogram (top). Dendrogram generated from identity-by-state distances; public camelina lines represented by their accession identifier (blue dendrogram leaves), study panel accessions with the “UNT” suffix (red dendrogram leaves). Admixture population genetics presented as a bar plot visualizing inferred genomic admixture for each camelina accession. Eight ancestral subpopulations were represented by 8 colors; each colored section in the bars represented the proportion of the accession's genome being inherited from the ancestral population indicated by color.

A total of 54 spring-type camelina accessions were selected to represent commercial and local varieties spanning diverse pedoclimates. To determine how representative the 54 accessions in the study panel (henceforth, UNT lines, Table S1) were of the available public accessions, a total of 200 camelina accessions (Li et al 2021) were analyzed using 2 approaches. Firstly, sequencing reads were aligned to the camelina DH55 reference genome (Kagale et al 2014) to identify a SNP variant matrix of 340,696 high-confidence SNPs. A PCA of this matrix (Fig. 1a) indicated that the study panel was representative of the wider population, with PC1 and 2 explaining 21% and 12% of the genetic variance, respectively, indicating a good representation of the total genomic diversity space. Secondly, to elucidate the pairwise genomic similarity in detail, a hierarchical clustering based on identity-by-state distances was generated aligning the resulting dendrogram tree with the results from an ADMIXTURE population genetics analysis (Fig. 1b). In this analysis, the public (Li et al 2021) and study panel camelina accessions were grouped by their genomic relatedness to infer which fractions of their genomes, based on SNP data, belong to which ancestral population. The ADMIXTURE analysis performed most effectively with 8 assumed subpopulations (Fig. 1b). Collectively this indicated that the study panel captured most of the genetic diversity observed in the larger public population and provided a representative resource to phenotype camelina morphophysiological responses to abiotic stress.

### Metabolic plasticity of the camelina panel to abiotic stress

To assess the response to abiotic stress, early stage drought and heat were imposed separately on 18-day-old plantlets for up to 10 days (Fig. S1). Drought was imposed by water withdrawal to 20% SWC and monitored thereafter (Fig. S1a). High temperature (32/25 °C day/night) was tracked using GDD (Fig. S1b). Plant development and growth were assessed by development stage 1 of BBCH scale (Martinelli and Galasso 2011), leaf length, and width (Fig. S2). All 3 traits demonstrated highly significant differences with treatment and line (Table S2a to c). Overall, heat stress advanced development, whereas drought stress had the opposite effect, together with significantly reduced leaf length and width. These results suggested that the imposed conditions caused a significant change in camelina development and merited further investigation of the plant's metabolism.

**Figure 2:**
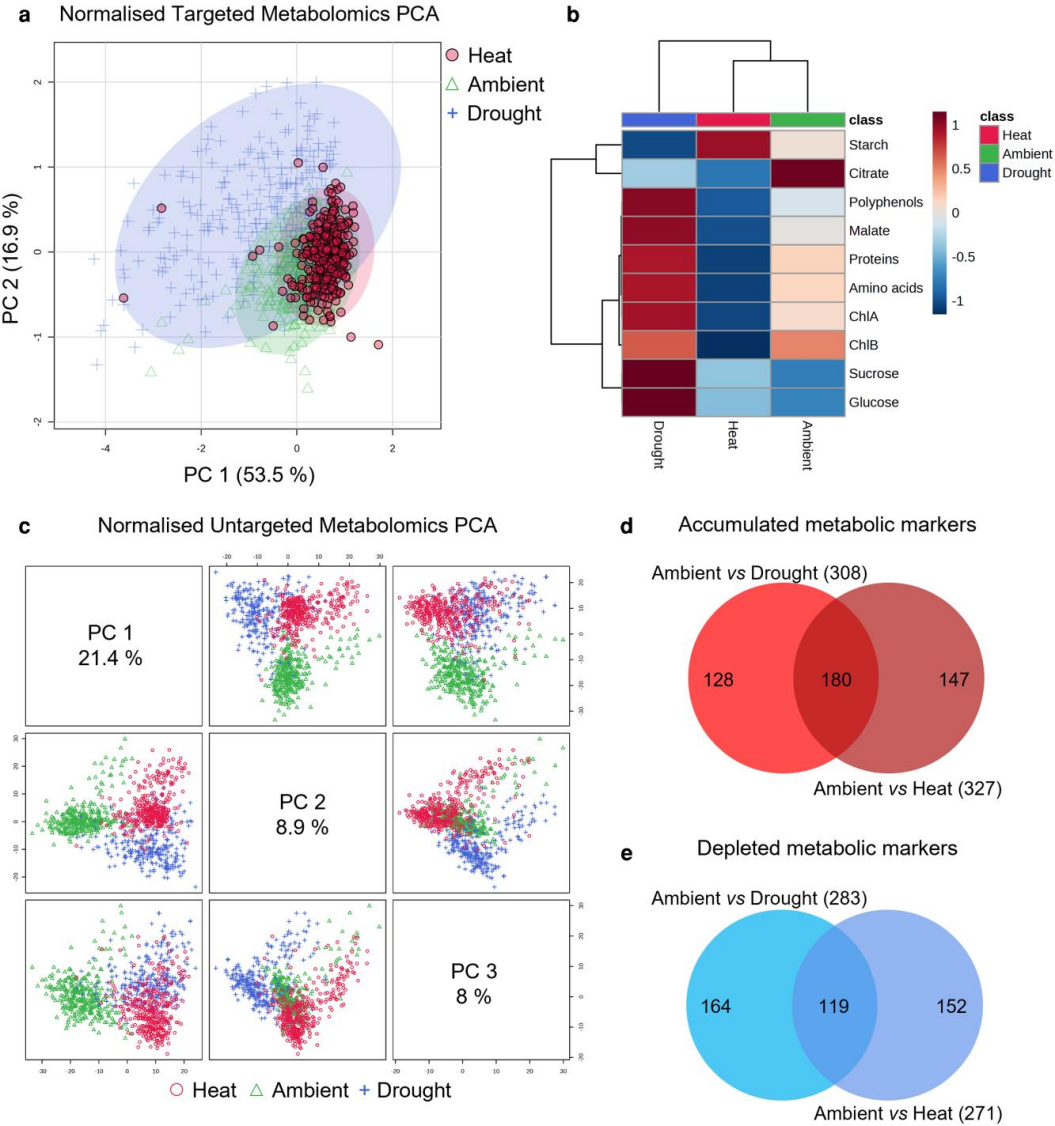
Metabolic changes in the camelina study panel in response to early stage drought and heat stress. a) PCA of 10 major compounds using normalized targeted data (median normalization, cube root transformation, and Pareto scaling). b) Heatmap clustering analysis (Pearson's correlation, Ward clustering). All markers presented statistically significant variations (ANOVA, *P* < 0.01 with adjusted FDR correction). Each column represents the conditions (ambient, drought, heat) averaged for all samples. Each row indicates the major compounds with relative intensity shown as a heatmap (blue, depleted; red, accumulated). c) PCA of normalized untargeted data (median normalization, cube root transformation, and Pareto scaling) showing PC1 vs PC2 vs PC3. a, c) Ambient, green triangle (Δ); drought, blue plus sign (+); heat, red circle (Ο). d–e) Venn diagrams show specific and common metabolic markers identified by volcano plots (Fold Change [FC] > 2; *t*-test *P* < 0.01, FDR corrected) that accumulated d) or decreased e) in response to drought or heat.

To do this, an (un)targeted metabolomics approach using complementary techniques was employed to profile primary and secondary leaf metabolites in response to the applied stresses. A PCA of the targeted (10 major) biomass compounds evaluated the global impact of drought and heat on camelina biomass (Fig. 2a). The PC1 vs PC2 score plot explained 70.4% of the total variance, showing that while ambient and heat stress conditions partially overlapped, drought stress was more scattered and had a more substantial impact, corresponding to separation along PC1. This indicated that camelina's central metabolism responded differently to each stress, with drought having a more substantial effect at early stage than heat. The heatmap clustering analysis (Fig. 2b) visualized relationships between samples and major compounds: all 10 biomass compound variables were statistically significant with respect to growth conditions (ANOVA, *P* < 0.01, False Discovery Rate [FDR]). Consistent with the PCA plot in Fig. 2a, drought stress clustered separately from ambient and heat stress conditions. Most of the major compounds analyzed accumulated under drought, except citrate and starch. Heat stress generally decreased compound content, except for starch, which increased, and soluble sugars (eg, glucose, sucrose), which remained stable.

**Figure 3:**
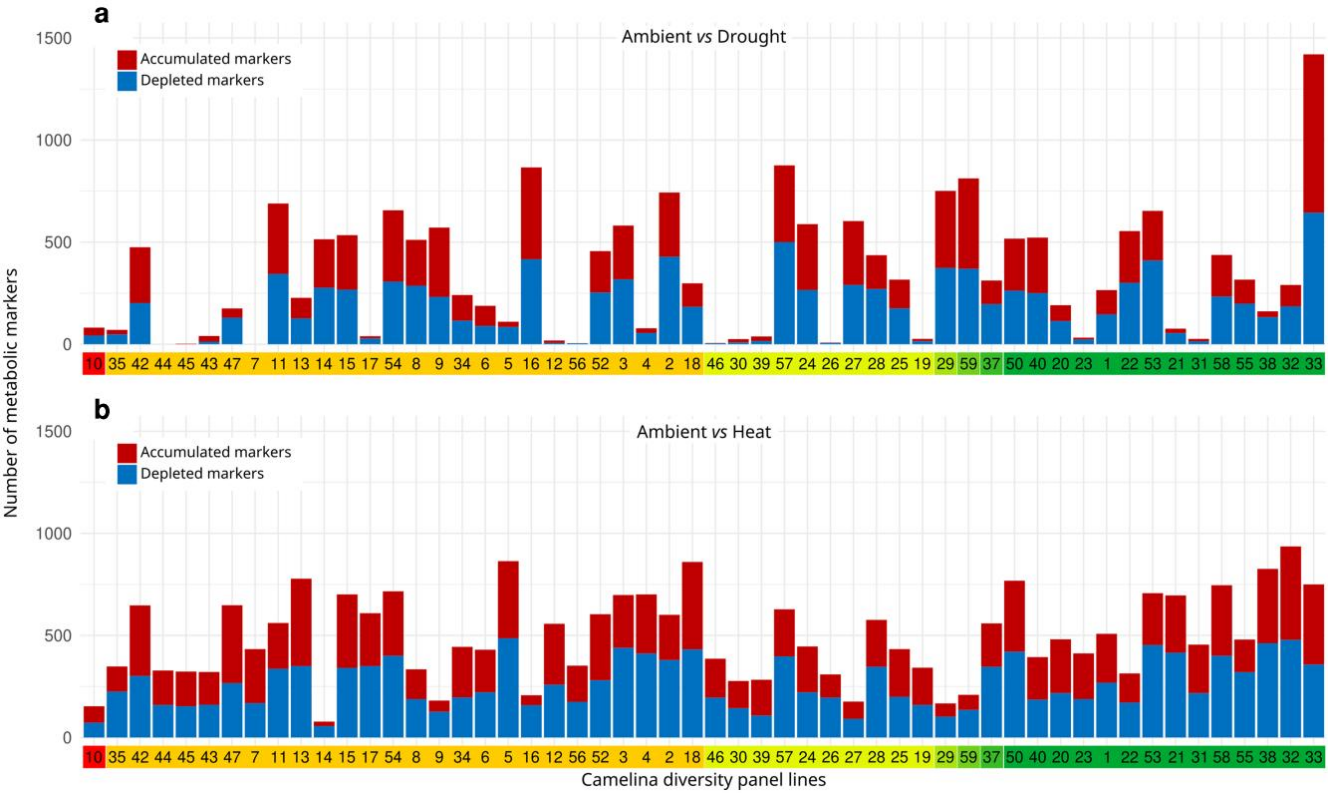
Response of metabolic markers to early stage abiotic stress. a) Drought and b) heat stress markers for each line in the camelina diversity panel plotted in the same order and color coded as determined by hierarchical clustering and admixture population genetics analysis shown in Fig. 1. Metabolic variables were qualified as markers if they were significantly accumulated or depleted when compared to ambient condition for a given camelina line (*t*-test, *P*-value < 0.05 FDR corrected; log_2_FC > 1 or log_2_FC < 1, respectively). FC, Fold Change.

Untargeted metabolomics further revealed clear shifts in the camelina metabolome between drought and heat stress (Fig. 2c; Fig. S3). The analysis identified 3,016 metabolomic features, of which 1,350 were unknowns, 1,446 had suggested annotated metabolites, and 220 matched with identified metabolites (Fig. S3a). Among these 3,016 features, which were normalized prior to statistical analyses, 2,545 were statistically significant regarding growth conditions (ANOVA *P* < 0.01, FDR; Fig. S3), indicating the robustness of the metabolomic dataset and the level of phytochemical diversity shifts. The global impact of drought and heat on camelina phytochemical diversity was assessed by PCA (Fig. 2c), where PC1 vs PC2 separated control, drought, and heat stress with a total variance of 30.3%. This indicated that the global metabolic profiles of camelina responded differentially to the type of stress. Since this metabolomic profile is mostly composed of secondary plant molecules, it further identified that stress-responsive, secondary metabolism is a better integrator to distinguish drought and heat impact than major compounds (Fig. 2a). In response to drought, 308 metabolic markers accumulated and 283 decreased, while in response to heat stress, 327 increased and 271 were depleted (Fig. 2d, e; Fig. S3b; Fold Change [FC] = 2, *t* test *P* < 0.01, FDR, between control and stress). For each stress, a balanced number of specific (128 and 147 for drought and heat, respectively) and common (180) accumulated markers and specific (164 and 152 for drought and heat, respectively) and common (119) depleted markers were observed. The accumulated and depleted markers for drought and heat stress were then stacked for each camelina line in the diversity panel (Fig. 3). This approach uncovered clear differences in the metabolic response of the different camelina lines to drought and heat stress. Some lines showed no or very low numbers of responding metabolic markers (eg, UNT7, 44, and 45 under drought. Fig. 3a; UNT10, 14 and 29 under heat, Fig. 3b). Other lines showed a pronounced overall metabolic response with high number of accumulated/depleted markers, eg UNT16, 33, 57, and 59 in drought (Fig. 3a) and UNT5, 18, 32, 38, and 50 in heat (Fig. 3b). Furthermore, some camelina lines had a strong response to drought but much less to heat (eg, UNT16), and conversely, individual lines had a strong response to heat and much less to drought (eg, UNT5), whereas other lines (eg, UNT33) had a large metabolic response to both drought and heat. Thus, adjustment of metabolism appeared to be an important part of the response of camelina to drought and heat stress, and there was substantial variation among lines suggesting plasticity in the camelina metabolic response to specific stresses.

To gain further metabolic insight, metabolic markers were putatively annotated using ClassyFire ontologies (Djoumbou Feunang et al 2016). Common markers (Table 1) included some increased phenylpropanoids (cinnamic and coumaric acids, flavonoids), carbohydrates (pentoses and phenolic glycosides), and benzenoids, along with several phenylpropanoids (phenolic acids and flavonoids) and organic acids (citric acid). Under drought, responses were diverse, with increased phenylpropanoids, phenolic acids and anthocyanins, amino acids (proline, histidine, and tyrosine derivatives), carbohydrates (phenolic glycosides), and lipids (terpene glycosides), as well as some decreased phenylpropanoids (chalcones and flavonoid glycosides), organic acids (succinic acid), benzenoids (benzoic acid derivatives), nucleotides, and lipids (FAs). Heat stress positively impacted phenylpropanoids (chalcones, flavans, and flavonoid glycosides), terpenes, benzenoids, and carbohydrates (O-glycosyl compounds), while negatively affecting some other phenylpropanoids (flavonoids, stilbenes, and phenolic acids), organic acids (TCA derivatives), lignans, and benzenoids (benzoic acids). Overall, metabolomics revealed a profound modification of primary (ie amino and organic acids, lipids) and secondary (ie phenylpropanoids, terpenes) metabolisms in responses to stress, with well-established markers.

**Table 1:** Summary of the metabolic response of the camelina diversity panel to early stage abiotic stress (drought and heat) measured in juvenile leaves.

	Common markers		Drought-specific markers		Heat-specific markers	
Metabolic pathways	Increased	Depleted	Increased	Depleted	Increased	Depleted
Alcohols and polyols	1	5	2	2	0	3
Alkaloids	0	2	0	0	4	2
Amino acids and derivatives	3	7	16	6	4	3
Benzenoids	**17**	5	5	**17**	13	12
Carbohydrates and derivatives	**28**	3	14	9	12	9
Hydrocarbon derivatives	1	2	0	2	0	0
Indoles	2	0	0	0	0	0
Lignans	1	2	3	0	1	12
Lipids and derivatives	3	7	11	11	8	3
Nucleotides and derivatives	2	3	2	11	2	2
Organic acids and derivatives	4	16	0	**17**	2	**17**
Organoheterocyclic compounds	5	10	14	6	4	8
Phenylpropanoids and polyketides	**26**	**36**	**34**	**17**	**35**	**28**
Terpenes	6	2	0	4	15	2
**Total annotated**	**94**	**58**	**64**	**54**	**85**	**65**

Stress responsive markers (common and stress specific) for individual metabolic pathways are shown, alongside the total annotated. Numbers in bold show highest increased and depleted markers.

### Camelina FA desaturation and remodeling in response to abiotic stress

**Figure 4:**
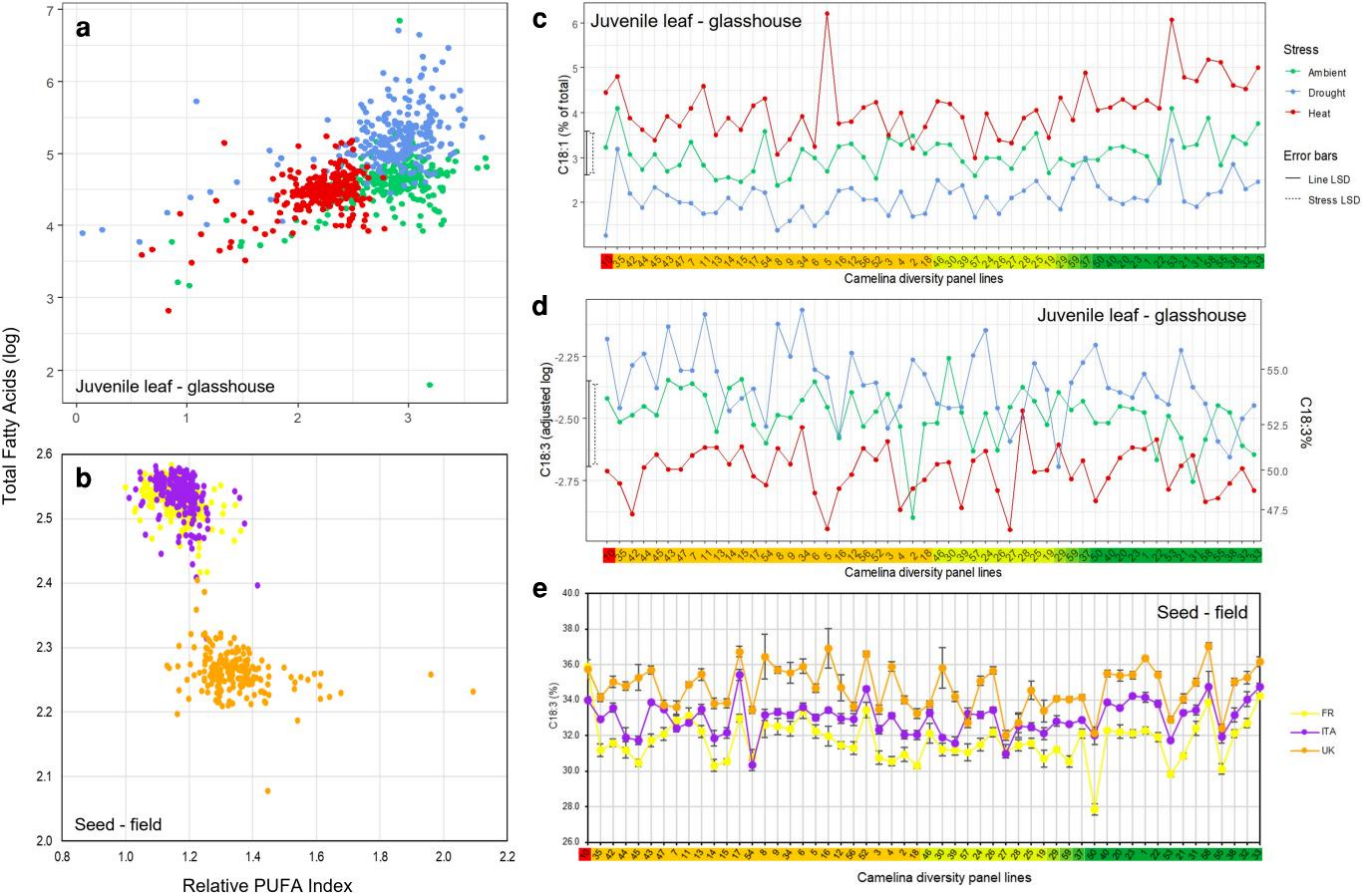
Leaf and seed fatty acid responses of the camelina diversity panel to early stage environment ambient (red), drought (green), and heat (blue) treatments and field trials in diverse pedoclimates (France [yellow], heat; Italy [purple], drought; United Kingdom [orange], temperate). Relationship between total fatty acids and relative polyunsaturated fatty acids (PUFA) ratio for individual lines/replicates leaves for each treatment a) and field grown seeds b). Leaf C18:1 c) and C18:3 (d, leaf; e, field seed) response to treatment for individual lines (*x* axis) plotted and color coded based on the distributions determined hierarchical clustering and admixture population genetics analysis shown in Fig. 1. Data were transformed to ensure assumptions of homogeneous variance hold and in the case of d) original scale shown on the right-hand side. Error bars illustrate least significant difference (LSD) between lines (solid black) and between stresses (dashed black). Interaction between stress and line was nonsignificant for leaf C18:3 d). e) Line mean ± standard error shown, Italy (ITA) and France (FR), N = 4; United Kingdom (UK), N ≤ 15.

Leaf FAs from the camelina early stage drought and heat stress experiment showed a significant impact of stress on total FA, relative polyunsaturated FA content (PUFA index), and C18:3 content (linolenic acid, Fig. 4a, c and d; Table S2d to f). C18:0 demonstrated a highly significant line effect and marginally significant effect of the stress (Table S2g), while C18:3 exhibited significant effects due to environmental stress and only marginal line differences. Lipid remodeling clearly demonstrated an impact of abiotic stress, and a response was observed across all accessions. The PUFA index reflected FA desaturation adjustment with heat as camelina remodeled membrane FA composition to maintain its fluidity. Heat decreased the calculated PUFA index to 2.2 compared to 2.8 under ambient conditions, while drought had the largest effect on total FA content, increasing the average amount to 182.4 µg/mg compared to 98.8 µg/mg under ambient. Plotting total FA and PUFA index (Fig. 4a) for each line clearly separated the 2 stress treatments and ambient. Oleic acid (C18:1) significantly declined with drought and increased with heat in all lines (Fig. 4c) and was the best indicator of environmental stress response in camelina. Within the population there was a line-specific response, with some lines showing significant changes with both treatments eg, UNT 5, 21, 31, 33, 38, and 58, while others had a stress-specific response, eg UNT22 and 37—responding to heat but not drought (Fig. 4c). Camelina remodeled its FA composition and content in response to environmental stress, and the extent of this remodeling was accession specific within the panel.

To further establish if leaf FA remodeling in response to abiotic stress reflected the response of mature plants in agronomic field trials, FAs were analyzed in mature seeds collected from the diversity collection grown in multilocation field trials (Fig. 4b, e; Table 2; Table S3). The three trials (France, Italy, and the United Kingdom; details in Supplementary  Methods) represented different pedoclimates and environmental challenges, namely climate during seed maturation, ie from 50% flowering to harvest and summarized as Italy—drought (GDD 828, precipitation 51.8 mm); France—heat (GDD 1,235, precipitation 224 mm); and the United Kingdom—temperate (GDD 721, precipitation 137 mm). For reference, long-term annual averages for precipitation and temperature at the trial locations are UK precipitation 702 mm, temperature 10.3 °C; France precipitation 772 mm, temperature 11.1 °C; and Italy 613 mm precipitation, 13.2 °C (details in Supplementary  Methods). Camelina seed lipid metabolism responded to environmental stress by remodeling FA, as indicated by the separation of Italy (drought) and France (heat) from the United Kingdom in the plot of total FAs and relative PUFA content (Fig. 4b), changing the levels of desaturation (Fig. 4e) at each trial location. Collectively, the controlled environment and multilocation field trials showed how camelina effectively utilized lipid metabolism and remodeling to respond to abiotic stress, and therefore, how FA composition can be used to identify stress responsive lines.

**Table 2:** Seed fatty acid composition for the camelina grown in multi-location field trials.

Trial	C16:0	C18:0	C18:1	C18:2	C18:3	C20:0	C20:1	C20:2	C20:3	C22:1
France	6.46 ± 0.020	2.59 ± 0.012	15.26 ± 0.095	18.08 ± 0.077	31.75 ± 0.092	1.72 ± 0.010	15.85 ± 0.041	2.01 ± 0.016	1.36 ± 0.011	3.23 ± 0.025
Italy	6.02 ± 0.019	2.65 ± 0.012	15.45 ± 0.077	17.23 ± 0.076	32.97 ± 0.074	1.62 ± 0.011	15.94 ± 0.044	1.91 ± 0.014	1.40 ± 0.008	3.16 ± 0.024
United Kingdom	5.91 ± 0.042	2.53 ± 0.012	15.03 ± 0.104	18.59 ± 0.091	34.70 ± 0.098	1.35 ± 0.008	14.12 ± 0.061	1.91 ± 0.012	1.39 ± 0.009	2.62 ± 0.020

Fatty acids as number of carbons:desaturations (% of total FAMES); France (N = 216); Italy (N = 216); United Kingdom (N = 672); results show average for all lines, ± standard error).

### Integrative analysis of the morphophysiological and metabolic response of camelina to abiotic stress

The impact of early stage abiotic stress in leaves was comprehensively assessed by measuring plant water content (PWC), Δ^15^N and Δ^13^C isotope discrimination, TAC, and relative chlorophyll content (SPAD; Figs. S4 and S5). On average, TAC decreased under both stress conditions, suggesting oxidative stress, with heat resulting in a stronger effect. Significant differences in PWC, Δ^13^C isotope discrimination and TAC were observed with respect to both treatment and line (Table S2h to j). Furthermore, leaf Δ^15^N significantly responded to heat treatment (Fig. S4b; Table S2k).

Overall, heat stress advanced development, whereas drought stress had the opposite effect, significantly reducing leaf length and width. Individual lines showed differing responses to abiotic stress (Fig. S2), for example lines UNT11, 12, 18, 21, 32, and 55 showed only a limited developmental response to stress, while others (eg, UNT6, 8, 34, and 56) had a much greater response. Drought stress had a significant impact on PWC (Table S2h, Fig. S4c). Individual lines lowered their PWC to less than 80%, eg UNT10, 11, 15, and 57, whereas others maintained PWC at control levels (87%) despite the drought treatment, eg UNT35, 44, 53, and 54 (Fig. S4c). PWC was maintained in heat stress plants, indicating that combined heat and drought stress did not occur in this experiment. As a further proxy measure of water availability, the degree of Δ^13^C isotope discrimination was determined in leaf samples (Fig. S4a). Drought resulted in a reduction in Δ¹³C in the study panel, which was significant for both treatment and line (*P* < 0.0001; Table S2i). Both the measurement of PWC and Δ¹³C indicated that the camelina study panel contained lines with variability in drought tolerance (Fig. S4). Resistance to environmental stress was also evaluated by measuring leaf chlorophyll content (SPAD) as an indicator of stress tolerance. The average SPAD measurements for the diversity panel grown in ambient conditions was 35.7 chlorophyll index units, heat 36.65 chlorophyll index units, and drought 44.09 chlorophyll index units (Fig. S5). These measurements indicated an increase in chlorophyll content following drought. However, not all the lines responded in the same way. For example, at the end of the experiment, UNT5 and 12 had the highest chlorophyll index values under ambient conditions, but UNT7 and 29 ranked highest following drought, while UNT12 and 25 ranked higher after heat. UNT31 consistently had the lowest chlorophyll index value in ambient, but under drought, UNT46 was lowest, and in heat UNT55. These results identified that chlorophyll content was differentially modulated in camelina lines to maintain photosynthetic performance under abiotic stress.

Cellular redox balance is crucial for abiotic stress response and acclimation. Metabolomics identified genotype-specific differences in antioxidants, and estimations of the redox balancing capacity of the studied camelina lines were made, determined as TAC in leaves (Fig. S4d). On average, TAC decreased under both stress conditions in the diversity panel suggesting oxidative stress, with heat resulting in lower TAC. Line-specific responses were highly significant effect (*P* < 0.0001; Table S2j). Under ambient conditions UNT28 exhibited the highest capacity and UNT45 the lowest, however UNT8 and 25 showed a higher overall TAC under drought. Some of the lines showed reduced TAC under both stresses, eg UNT11, 40, and 58; while others, eg UNT13, 56, 39, and 22, only responded significantly to drought; and some, eg UNT4, 14, 16, and 54, only to heat, indicating that the study panel had significant variability, in terms of cellular redox response, to abiotic stress.

**Figure 5:**
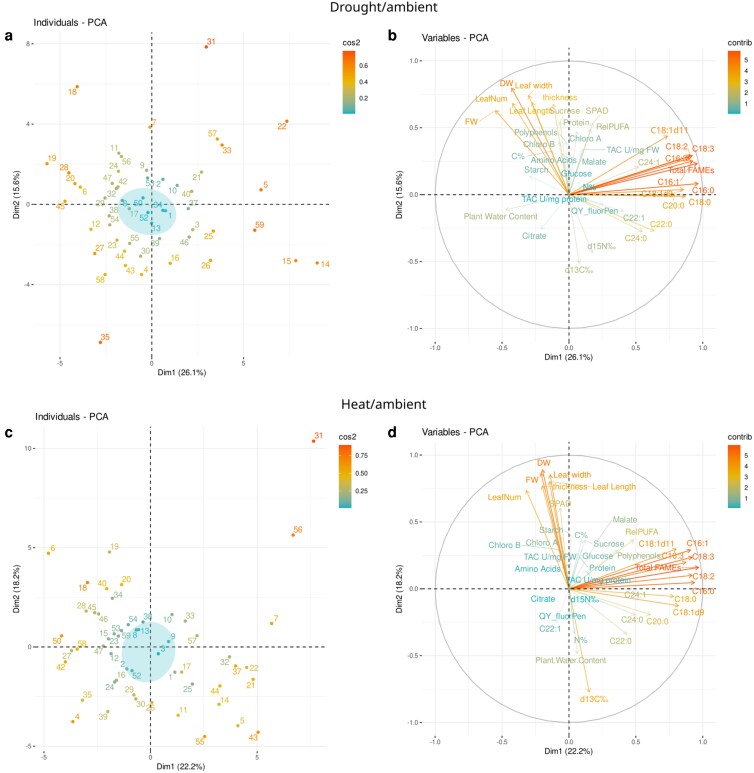
PCA on measured traits from the camelina diversity panel early stage abiotic stress study. Drought over ambient a, b) and heat over ambient c, d) for the individual lines; a and c), colors represent cos2 values as a proxy for the quality of representation in the 2D space (ratios were scale-normalized before performing the PCA; blue shading highlights “unresponsive” lines); and the measured variables b and d), colors represent the contribution of the variables to the PCA: leaf fatty acids, chlorophyll content (SPAD), stable isotope (Δ^15^N, Δ^13^C), phenology (BBCH scale, stem thickness, leaf number, width, length), SWC, fresh and dry weight, plant water content, and major biomass components: glucose, sucrose, starch, malate, citrate, total polyphenol content, free amino acid, total protein content, chlorophyl a and b).

The collected data traits, ie morphophysiological markers comprising TAC, leaf FA, SPAD, stable isotope discrimination, phenology (BBCH), plant development (stem thickness, leaf width, number and length, SWC, PWC, and plant fresh and dry weight), were combined with FA and targeted metabolites for an integrated analysis by PCA for drought and heat stress (Fig. 5). Camelina showed a clear developmental, physiological, and biochemical response to abiotic stress. In response to heat and drought, plant development and FA contributed strongly to this analysis (Fig. 5b and d, illustrated by red and orange arrows). It was interesting to note that Δ¹³C discrimination had a larger impact in heat than drought, despite SWC and PWC being maintained at ambient levels in heat stress. However, the line distributions were different in response to each stress (Fig. 5a and c). Metabolic, biochemical, and physiological parameters (eg citrate, malate, polyphenols, chlorophyll, TAC, stable isotope discrimination, and plant water status) identified “responsive” lines, eg UNT5, 18, 22, 28, 31, 35, 59 (red), and “unresponsive,” eg UNT lines 1, 8, 13, 34, 50, and 52 (blue; Fig. 5a; Table S4a) to drought. The distribution of lines in heat showed responsive, eg UNT18, 31, 50, 56 (red/orange), and unresponsive lines, eg UNT3, 8, 9, 13, 38, 52 (blue; Fig. 5c; Table S4b). Abiotic stress-responsive lines common to heat and drought included UNT18 and 31, while common unresponsive lines were UNT8, 13, and 52. However, there was little commonality in these lines in terms of accumulation/depletion of metabolic markers in response to stress (Fig. 3) or genetics (Fig. 1).

### Connecting plasticity in camelina stress responses to agronomic performance

Multilocation field trials in diverse pedoclimates were used to assess the response of mature plants to challenging environments and assess the agronomic performance of the camelina panel. Yield, plant density, stand height, TGW, and seed oil content/composition were measured (Table 2; Figs. S6 and S7; Tables S3 and S5) in the diversity panel at the 3 locations: Italy, drought; France, heat; and the United Kingdom, temperate. Seed oil content and composition, together with TGW across lines, remained relatively consistent in all 3 field trial locations, whereas other phenotypic traits (yield, height, density) were clearly different (Figs. S6 and S7). Overall, the diversity panel had the highest yield in Italy; the camelina lines responded well to drought, whereas in France (heat) the lines grew taller, accumulating biomass with the increase in temperature and sufficient precipitation. In response to the different environments, the coefficient of variation (CV; Table S6) estimated for TGW, oil content, and yield at the 3 locations indicated limited variation for oil content (4.4 to 5.1) and TGW (12.5 to 13.9), while the panel CV for yield was high and more variable (22.4 to 57.2). Most notably, yield in Italy (drought) appeared to be much more robust among the lines and demonstrated a different relationship to other traits when compared to France (heat) and the United Kingdom (temperate) (Fig. S6f). Assessment of agronomic performance distinguished individual lines in their response to the local climates (Table S4, Fig. S6). UNT57 had the highest TGW at all 3 trial locations, whereas UNT33, 21, and 59 had the lowest. As indicated by the CV (Table S6), TGW was stable across environments, while yield and seed oil content were more variable and sensitive to genotypic × environment (G × E) interactions at all locations. Analysis of mature seed FA content showed a strong correlation in composition across the 3 trial locations (Fig. S7). Genotypic trends in FA composition were relatively consistent across the 3 trials. In particular, the panel lines aligned similarly in the first 2 PC projections across the trial locations. PCA of seed FA (Fig. S7b) identified responsive (eg UNT12 15, 19, 21, 22, and 55) and unresponsive (eg UNT46 and 54) lines consistently across the 3 locations. Location-specific differences in PC1 appeared to be driven by C20:1 (United Kingdom temperate different to France and Italy abiotic stress) and C20:0 (France [heat] in contrast to the United Kingdom [temperate] and Italy [heat]).

**Figure 6:**
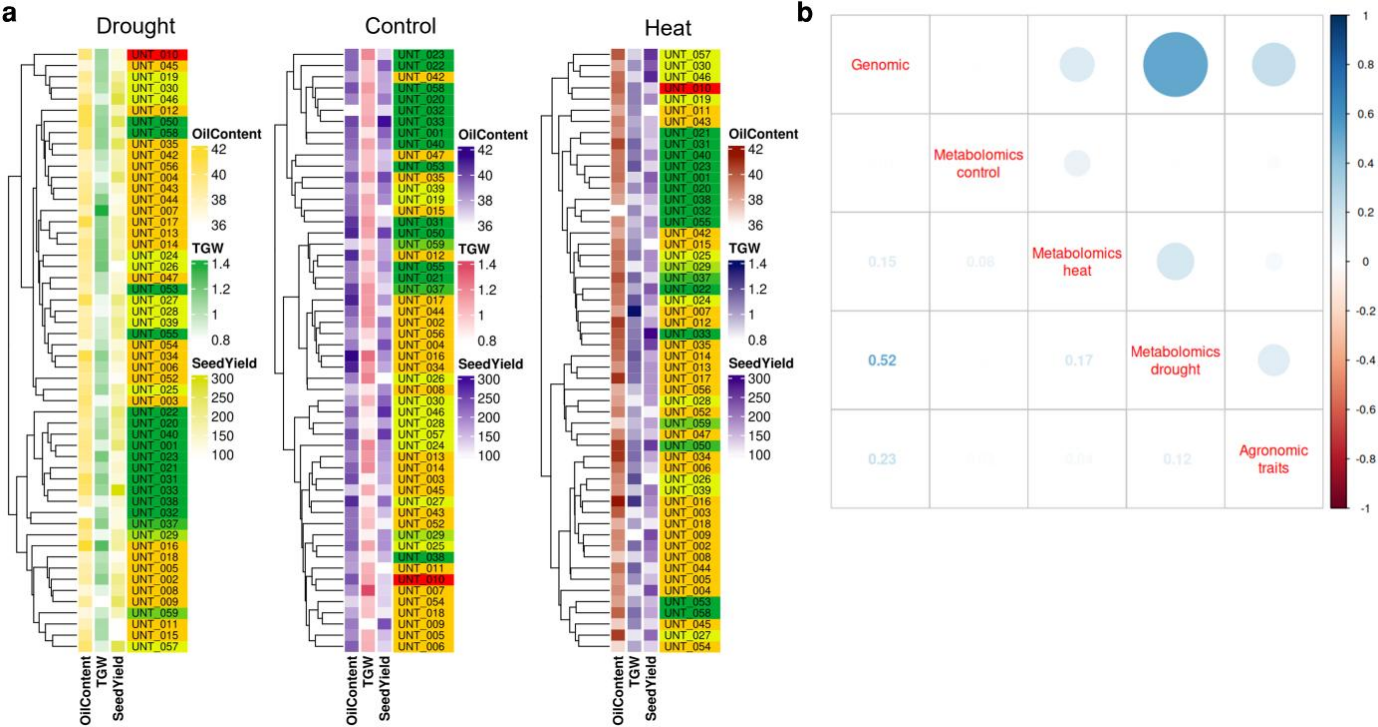
a) Relationship between metabolomic clustering and agronomic performance under different environmental conditions. For each of the 3 glasshouse conditions tested (control, drought, and heat stress), genotypes (color coded after Fig. 1) were clustered based on their metabolomic profiles using hierarchical clustering. Agronomic parameters (seed yield, g/m^2^), TGW (g), and seed oil content (% of seed weight) were represented as color gradients aligned with the dendrogram tips. b) Comparison of dendrogram similarity between phylogenetic, agronomic, and metabolomic data. Correlation plot showing pairwise Baker's Gamma correlation coefficients between dendrograms derived from phylogenetic, agronomic, and metabolomic data (from early stage experiments: control, drought, and heat stress). Each coefficient quantified the structural similarity between 2 dendrograms, with values ranging from −1 (complete discordance) to 1 (perfect agreement). Numerical values of the coefficients are shown in the lower triangle. The upper triangle displays a graphical representation of the correlation strength.

As discussed above, the studied lines showed significant differences in their metabolic, physiological, and developmental responses to heat and drought. Table S4 illustrates the different stress-responsive strategies deployed by these lines, eg in drought UNT31 was highly responsive in the combined PCA but had a low ranking in accumulated/depleted markers, whereas UNT22 was also responsive but accumulated/depleted substantial numbers (∼600) of metabolic markers (Table S4a). The distribution for unresponsive (Fig. 6) lines was similar, with individual lines, eg UNT50 and 31, showing a range of accumulated/depleted metabolic markers. The pattern was repeated for heat stress (Table S4b). PCA identified unresponsive, eg UNT8 and 13, and responsive lines, eg UNT56 and 18 with divergent metabolic marker responses. Our approaches provided a unique opportunity to evaluate how these differing response patterns could be related to agronomic performance. Looking closely at the stress (un)responsive subset, lines UNT22 and 59 were responsive to drought (PCA) with large numbers of accumulated/depleted metabolic markers and had a very low TGW across all 3 trials. In this case, UNT22 and 59 showed a strong stress response, which did not translate into good field performance. In contrast, UNT5 and 28 were also responsive to drought (PCA) but did not accumulate/deplete large numbers of metabolic markers and yet performed well in the field. This case illustrates how lines UNT5 and 28 maintained field performance via physiological adaptation rather than metabolic adjustment (as determined by metabolic profiling). Field performance (TGW) differentiated lines unresponsive to drought (PCA) and with a similar metabolic marker responsiveness, eg UNT34 and 13. Indeed, UNT8 performed very well in the field (TGW and oil content), was unresponsive (Fig. 6), and had a moderate metabolic marker drought response. Metabolic responses in UNT8 might have enabled the plant to prosper in response to both drought and heat. Heat stress produced a similar plasticity of responses across the measured parameters for early stage stress (PCA and metabolic markers; Table S4b). Some lines performed well in the field (TGW), eg UNT8 and 13, and were unresponsive to heat (PCA), whereas others were responsive to heat and performed badly in the field, eg UNT31 and 50. These results indicate that crop agronomic performance was not necessarily associated with robust metabolism and therefore low plasticity. On the contrary, high plant plasticity with dynamic metabolism could therefore be coupled with improved agronomic performance, identifying metabolic plasticity as an indicator of resilience to abiotic stress and field crop performance in changing climates. Further analysis was performed to establish a relationship between the metabolic clustering presented in each of the early stage conditions applied—control, drought stress, and heat stress—and 3 main measures of agronomic performance—TGW, seed yield, and oil content (Fig. 6). However, global metabolomic performance in each of the conditions applied did not correlate with agronomic performance (Fig. 6a). To evaluate the concordance between genotype and phenotype-related data, Baker's gamma correlation coefficients were computed between the dendrograms obtained from genomic, agronomic, and condition-specific metabolomic data (Fig. 6b). The highest correlation was observed between the genomic dendrogram and the metabolomic clustering under drought stress (0.52), indicating a moderate structural similarity and the possibility that camelina is inherently adapted to drought. A weaker, but still positive, correlation was also found between genomic and agronomic clustering (0.23). The other comparisons showed lower correlations: between metabolomics under drought and heat stress (0.17), between heat stress metabolomics and genomics (0.15), between agronomic and drought metabolomics (0.12), and finally between metabolomics under heat and control conditions (0.08).

## Discussion

### Camelina genetic diversity and the potential for crop improvement

An understanding of how available genetic diversity in camelina underpins its stress resilience is vital for developing a location-specific breeding strategy for further crop improvement and to understand the plasticity of abiotic stress responses. Camelina (Brassicaceae) has a hexaploid genome structure generated by the merger of 3 diploid genomes (Mandáková et al 2019; Mandáková and Lysak 2022; Bird et al 2025). Reports in the literature have generally suggested low levels of genetic diversity in camelina compared to other oilseed crops (Chaudhary et al 2020). This genetic paucity has been recognized with approaches developed to address the diversity challenge (Blume et al 2023). However, it is noteworthy that detailed explorations of large camelina populations (see Luo et al 2019) have demonstrated enough genetic diversity for developing new cultivars with desirable agronomic traits. Moreover, abiotic stress studies (Čanak et al 2020; Smith and Lu 2024) have shown diverse responses of different camelina genotypes. However, much of camelina's varietal diversity has been lost, particularly when European farmers shifted their interest from camelina to rapeseed and sunflower. Therefore, current publicly available germplasm collections are almost entirely composed of previous cultivated varieties and have a low genetic diversity and a high proportion of admixture. Many camelina breeding lines and cultivars were collected from the Russia-Ukraine region (Vollmann et al 2007), the common origin area of camelina (Brock et al 2018). In this study, we demonstrated that the collected camelina panel adequately represented the publicly available camelina germplasm. Furthermore, sequencing analysis identified the presence of 8 subpopulations, and PCA based on SNPs revealed that the study panel provided a good representation of the global camelina genomic diversity space. Knowledge of population structure and genetic diversity within this panel enabled a detailed investigation of camelina genotype-specific abiotic stress responses.

### Camelina abiotic stress metabolic response

**Figure 7:**
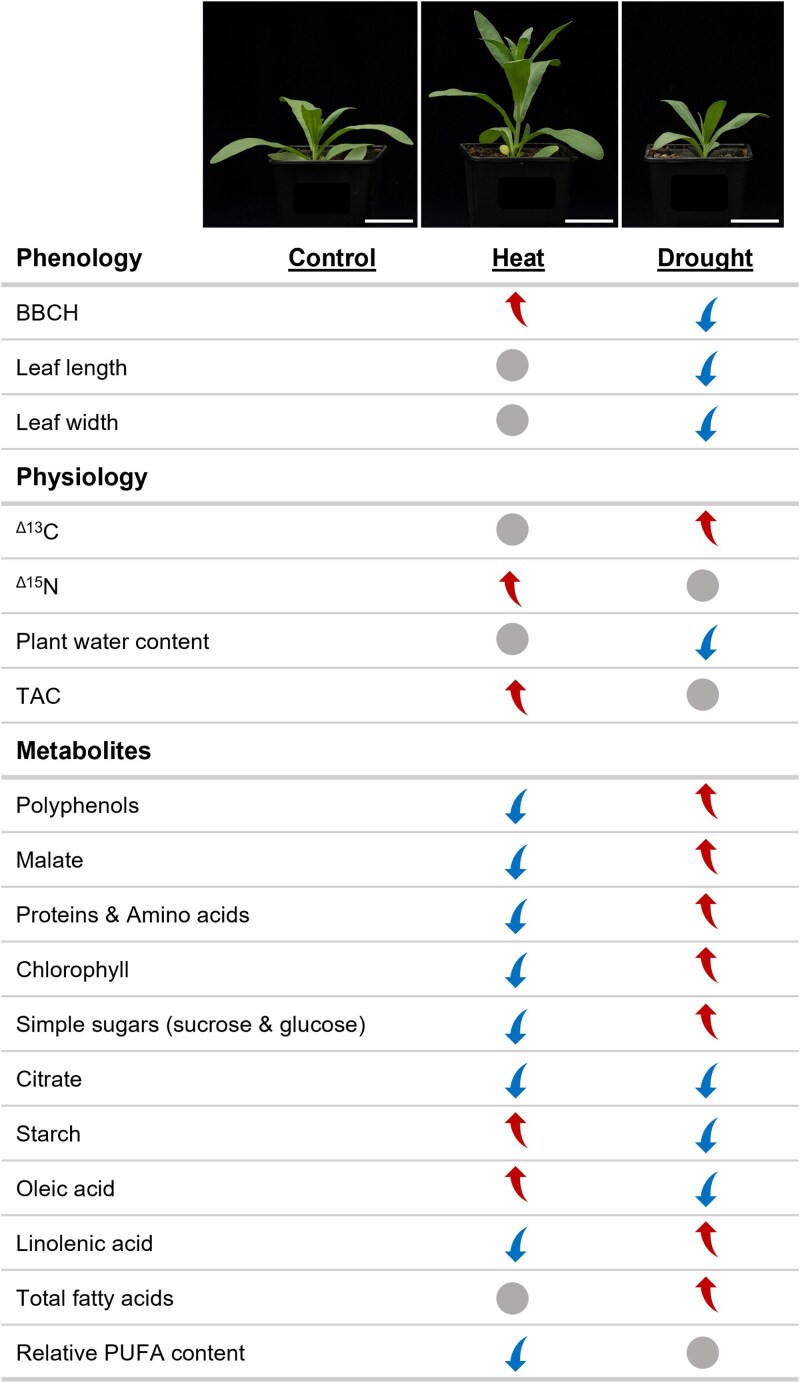
Summary of the multilevel stress responses *Camelina sativa* deploys to abiotic stress (heat and drought). Selected variables are shown to indicate the overall morphophysiological (phenology, physiology and metabolic) response shown by juvenile leaves to treatment, as indicated in earlier figures (Figs. 2 to 5; Figs. S2 and S4). These responses may vary between lines. Red arrow, increase; blue arrow, decrease; grey dot, nonsignificant change. BBCH, scale of phenological growth stages; Δ^13^C, and Δ^15^N leaf stable isotope analysis; TAC, total antioxidant capacity; PUFA, polyunsaturated fatty acids. Scale bar = 5 cm.

The imposition of heat and drought stress on the camelina panel at a juvenile stage of development had a significant impact on development and growth (Fig. 7). Plants were smaller and grew slowly with drought, while heat accelerated the developmental stages. These responses significantly differed between lines, indicating that the existing genetic diversity of camelina supports a portfolio of morphophysiological adaptation strategies. Although responses of this type might be anticipated to heat and drought stress (Čanak et al 2020; Nadakuduti et al 2023; Smith et al 2024), this study demonstrated the impact of abiotic stress at the juvenile stage across a diversity panel and the translation of this robustness to different field environments. Developmental age is a strong determinant of stress responses in plants (Rankenberg et al 2021). With aging, plants alter their organ morphology, sink–source balance, and chemical composition, including changes in redox status, which collectively influence how abiotic stress signals are perceived and processed. In this study, initial investigations focused on the physiological and metabolic response to drought and heat stress in young leaves before the initiation of inflorescences to understand the plasticity of stress resilience early in juvenile development. Drought had a significant impact on plant water relations and gas exchange. The reduced level of carbon isotope fractionation (Δ^13^C) discrimination was consistent with crop drought responses (Avramova et al 2019). Plant Δ^15^N reflects the values of external N sources and ^15^N/^14^N fractionations, which occur during assimilation, transport and loss of N (Robinson 2001). Δ^13^C and Δ^15^N are often used to determine drought responses in crops, and as expected, Δ^13^C was a good indicator of drought stress (reflecting changes in stomatal opening), while Δ^15^N separated ambient and heat stressed plants. Heat stress impacted growth and development, leaf nutrient status, uptake, and translocation (Mishra et al 2023). Likewise, TAC in young camelina leaves varied significantly and was typically reduced by heat and drought. Juvenile camelina plants demonstrated significant plasticity in response to abiotic stress. A significant part of this response relied on metabolic adjustment, likely triggered by a network of signaling cascades, mediated by reactive oxygen species (Choudhury et al 2017) and lipid signals (Sharma et al 2023).

Metabolic adjustment was central to the abiotic stress tolerance and plasticity observed. Targeted leaf metabolite analysis of juvenile leaves identified an asymmetric response to heat and drought. For example, citrate, malate, sucrose, glucose, proteins, and amino acids declined with heat and increased with drought, whereas starch increased with heat and declined with drought. Typically, abiotic stress impacts growth earlier than photosynthesis, which leads to energy imbalance and favors the accumulation of ROS. Plants respond by shifting to protective mechanisms, which include the accumulation of various metabolites (Zandalinas et al 2022), including antioxidants (Decros et al 2019). Major carbohydrates such as glucose, fructose, and sucrose have been shown to accumulate and starch to decrease in many species in response to drought (reviewed in Zandalinas et al 2022), reflecting reduced plant growth (decreasing the demand for primary metabolites) combined with a shift toward soluble compounds contributing to osmotic adjustment (Hummel et al 2010; Hildebrandt 2018; Heinemann and Hildebrandt 2021). Heat led to a reduction in levels of primary metabolites in camelina, a response that reflected the effect of heat on carbon utilization and distribution in plants. Together, photosynthesis and respiration underpin the plant metabolic response to heat. Photosynthetic thermotolerance typically results from genotypic variation in several essential processes, eg leaf net photosynthesis, transpiration stomatal conductance, and the thermostability of photosystems and electron transport. Other processes impacted include cell membrane thermostability (lipid remodeling), nonphotochemical quenching protection, heat shock protein production, and the thermal liability of Rubisco activase. Heat typically modifies sucrose metabolism and increases rates of respiration. Whereas phloem sucrose export by sucrose exporters and/or import by sucrose transporters are inhibited in heat-stressed leaves, which further results in starch accumulation (Braun et al 2014). The opposing rearrangements of starch metabolism with heat stress highlight the plasticity of starch metabolism and reflects other factors, eg tissue type developmental stage and growth (Thalman and Santelia 2017).

For the initial exploration of metabolic plasticity in the camelina panel, CVs were calculated for 10 targeted leaf biomass compounds in response to heat and drought (Table S5). CV is a measure of dispersion around the mean within a dataset, which allows the exploration of phenotypic variability. In control conditions, the highest CV values (indicating a large dispersion among replicates and, perhaps, plasticity within the study panel) were found for glucose, chlorophyll *b*, and starch, while the lowest CV values (low variability) were found for malate, polyphenols, and amino acids. Under abiotic stress, CV values of several major leaf compounds changed. CV of starch increased with drought and declined with heat; chlorophyll *b* and sucrose declined with both stresses; and glucose declined with heat. In contrast, CV values for polyphenols, amino acids, and chlorophyll *a* remained constant regardless of the treatment. The camelina panel thus displayed substantial variation in metabolic plasticity, which was modulated by abiotic stress.

Secondary metabolite multifunctionality can provide a better understanding of plant stress responses. The metabolomic analyses presented here identified the integral accumulation of plant secondary metabolites in the response of camelina to challenging environments. Metabolomic profiling showed that central metabolism of camelina responded differentially to the type of stress, with a more substantial effect of drought than heat. Untargeted analysis further confirmed the differential response to the different types of stress and showed that stress-responsive secondary metabolism distinguishes drought and heat treatments better than major compounds of plant biomass. In line with our results, seed metabolomic characterization of 6 field-grown camelina genotypes showed high levels of plasticity depending on year and genotype (Boutet et al 2022). Furthermore, high levels of plasticity in primary metabolites, including some sugars and major storage compounds such as FAs, proteins, and most lipid classes, were found in 2 genotypes (Boutet et al 2022). Classically, stress compounds such as proline, γ-amino butyrate, polyamines, and branched-chain amino acid metabolism underpin the response of camelina to stress conditions. The biosynthesis of polyphenols (eg phenolic acids, flavonoids, stilbenoids, and lignans) by camelina might alleviate the impact of stress-induced oxidative stress and the overproduction of ROS associated with environmental perturbation. Excess levels of ROS are a significant challenge, and camelina has multiple strategies to mitigate their impact. For example, estimates have indicated that camelina seeds have oxidative pentose phosphate pathway fluxes that exceed the demand for NAD(P)H for biosynthesis and are larger than those measured in other systems (Carey et al 2020). It is possible that high levels of NAD(P)H in camelina could contribute to its antioxidative capacity.

Plant cells further respond to stress by undertaking remodeling of cellular lipid species to maintain cell integrity and functionality, exchanging FA (eg C18:1, C18:2, and C18:3) in membranes to maintain fluidity (Upchurch 2008). Camelina responded to heat and drought stress by significantly changing leaf FA composition; oleic acid showed a striking line-specific increase in response to heat and decrease in drought; while linolenic acid declined with heat, indicating reduced membrane desaturation. These responses, mediated by FA desaturases (eg FAD2, FAD3) that are directly regulated by temperature, are typical of the cells' strategies to cope with abiotic stress (Gishini et al 2025). However, the response of lipids to drought is less well characterized. FAs are often remodeled in response to drought with levels of C18:3, C18:2, and C18:1 responding to maintain membrane fluidity. The decline in membrane fluidity is a disadvantage to crop growth under drought stress, because membrane fluidity plays an important role in stabilizing cellular metabolism and function. This study identified C18:1, along with C18:3 in seeds, as diagnostic markers for stress. This is consistent with recent camelina QTL mapping results in which markers associated with C18:1 were persistently detected in a diversity panel grown in multiple environmentally diverse locations. Furthermore, variation in C18:3 was associated with environmental temperature at the locations (Decker et al 2025). The temperature response of monounsaturated FAs (C18:1) is often species and tissue specific, reflecting the regulatory control of stearoyl-acyl carrier protein desaturase, which catalyzes the first desaturation step leading to C18:1. Responses of C18:1 to stress, eg heat and drought, likely reflect the combined regulatory control of Acyl-ACP thioesterase genes FATB and FATA (Byfield and Upchurch 2007). Remodeling FAs in molecular lipids is also achieved via the activity of lipases, eg monoacylglycerol lipase (MAGL), involved in triacylglycerol breakdown. Expression of MAGL gene candidates has been correlated with the response to abiotic stress and oil assembly pathways (Kumar et al 2025). Other lipase candidates responsive to heat include HEAT INDUCIBLE LIPASE1, which digests monogalactosyldiacylglycerol to produce 18:3-free FA. Like many crops (Higashi and Sato 2019), camelina has multiple remodeling activities to re-orientate the cellular lipidome and maintain cellular functionality and integrity. Lipid remodeling is a complex process of interacting activities, and a greater understanding of climate-related lipid remodeling will guide the development of lipid markers for resilient germplasm selection.

The efficiency with which camelina can reconfigure metabolic networks, resume active growth, and establish a stress-tolerant state varied across the panel. The application of correlative approaches within metabolomics enabled the identification of metabolites (biomarkers) associated with stress-responsive states. Biomarkers of this type are often referred to as metabolic markers when derived from metabolite concentrations and have been recognized and used for the selection of optimized germplasm (Lamari et al 2018; Xu et al 2025). Camelina lines within the panel displayed a variety of response mechanisms, which included physiological adaptation and integrated cellular or molecular responses. For example, lines displaying a large range of metabolic strategies: some lines (eg UNT33, 32) had a significant metabolic stress response, while the lines with specific metabolic responses to drought and heat (eg UNT16, 5, 44, 45, 10) had only small amounts of adjustment. Drought was clearly associated with the broadest range of accumulated/depleted metabolic markers (1,400 for UNT33 and zero for UNT26, 46, 56, 45, 7 and 44), which was nonetheless not correlated with field performance. Combinatorial assessment of the impact of stress on juvenile plants could identify responsive lines with large metabolic plasticity and good field agronomic performance, eg UNT28 and 18, but also lines categorized as unresponsive (eg UNT8) that also performed well in field environments. The moderate correlation observed between metabolomic profiles under drought stress (but not heat) and genomic structure (0.52; Fig. 6b) suggested that genetic background only partly explained the metabolic response to drought. This indicated that some pathways or metabolites involved in drought tolerance were under genetic control and shared among related genotypes. This might also indicate that camelina is particularly well adapted to drought rather than heat, as indicated by the performance of the diversity panel in a field trial located in France. The lower correlation between genomic and agronomic clustering (0.23) highlighted the fact that agronomic traits were probably influenced by more complex factors, such as plasticity or G × E interactions, which were not fully captured by genomic variation alone. Similarly, relatively low correlations between metabolomic profiles under different stress conditions (drought vs heat) showed that metabolic responses were quite specific to each environment, with different pathways activated depending on the stress, once again showing the plasticity of camelina to stress. The metabolic signatures thus provided a method to characterize this variation and identify specific camelina lines (Fernandez et al 2016; Stasnik et al 2024), thereby underscoring the crucial role of metabolomic studies in advancing research on orphan crops such as camelina. Despite its perceived lack of genetic diversity, camelina was nonetheless able to display remarkable plasticity in response to stress, providing an interesting leverage in crop improvement programs to identify improved ideotypes for specific pedoclimatic conditions.

Over recent years, research efforts have been dedicated to improving seed yield and agronomic traits in camelina (Blume et al 2022, 2023). One approach was to tap into previously unexploited germplasm and characterize the population for traits, eg juvenile abiotic stress tolerance, that will future-proof camelina for extreme climates, as shown in this study. After first examining and demonstrating the line-specific stress response in the diversity panel, we assessed how the measured diversity translated into agronomic performance in replicated multilocation field trials with different environmental challenges. Trait analysis of the diversity panel grown across the 3 trial locations demonstrated that the population contained lines capable of performing in different climates. The study confirmed the need to identify abiotic stress plasticity in camelina in combination with field testing. To capture both approaches, scaled performance parameters were established, eg TGW scaled by T_min_ (°C, at 50% flowering) linking environment measures that reflect heat or drought stress and crop traits. This approach could be deployed within a breeding strategy to develop climate resilient location-specific varieties. Furthermore, it could also be adopted for other ancestral crops as they become increasingly adopted by growers seeking to improve the climate resilience and diversity of their cropping systems.

## Accession numbers

Further information related to the enzymes mentioned in this manuscript can be found in https://fatplants.net/home.

IN A NUTSHELL Assessment of early stage abiotic stress in *Camelina sativa* identified line-specific stress-responsive morphophysiological signatures with a connection to crop productivity in field conditions.

## Supplementary Material

kiag052_Supplementary_Data

## Data Availability

The data relating to the experiments described in this manuscript can be found in the Camelina Plant Adaptation Hub (https://www.camelina-hub.org/). Metabolomics raw data are available at https://www.ebi.ac.uk/metabolights/MTBLS9839, and the processed data can be found in https://doi.org/10.57745/ZAFPL9.

## References

[kiag052-B1] Alexander DH, Novembre J, Lange K. 2009. Fast model-based estimation of ancestry in unrelated individuals. Genome Res. 19:1655–1664. 10.1101/gr.094052.109. 19648217 PMC2752134

[kiag052-B2] Alwood JW et al 2021. Unravelling plant responses to stress—the importance of targeted and untargeted metabolics. Metabolites. 11:558. 10.3390/metabo11080558. 34436499 PMC8398504

[kiag052-B3] Avramova V et al 2019. Carbon isotope composition, water use efficiency, and drought sensitivity are controlled by a common genomic segment in maize. Theor Appl Genet. 132:53–63. 10.1007/s00122-018-3193-4. 30244394 PMC6320357

[kiag052-B4] Bird KA et al 2025. Allopolyploidy expanded gene content but not pangenomic variation in the hexaploid oilseed *Camelina sativa*. Genetics. 229:1–44. 10.1093/genetics/iyae183. 39545504

[kiag052-B5] Blume RY, Kalendar R, Guo L, Cahoon EB, Blume YB. 2023. Overcoming genetic paucity of *Camelina sativa*: possibilities for interspecific hybridization conditioned by the genus evolution pathway. Front Plant Sci. 14:1259431. 10.3389/fpls.2023.1259431. 37818316 PMC10561096

[kiag052-B6] Blume RY, Rakhmetov DB, Blume YB. 2022. Evaluation of Ukrainian *Camelina sativa* germplasm productivity and analysis of its amenability for efficient biodiesel production. Ind Crops Prod. 187:115477. 10.1016/j.indcrop.2022.115477.

[kiag052-B7] Bolger AM, Lohse M, Usadel B. 2014. Trimmomatic: a flexible trimmer for Illumina sequence data. Bioinformatics. 30:2114–2120. 10.1093/bioinformatics/btu170. 24695404 PMC4103590

[kiag052-B8] Boutet S et al 2022. Untargeted metabolomic analyses reveal the diversity and plasticity of the specialized metabolome in seeds of different *Camelina sativa* genotypes. Plant J. 110:147–165. 10.1111/tpj.15662. 34997644

[kiag052-B9] Braun DM, Wang L, Ruan Y-L. 2014. Understanding and manipulating sucrose phloem loading, unloading, metabolism, and signalling to enhance crop yield and food security. J Exp Bot. 65:1713–1735. 10.1093/jxb/ert416. 24347463

[kiag052-B10] Brock JR, Dönmez AA, Beilstein MA, Olsen KM. 2018. Phylogenetics of Camelina Crantz. (Brassicaceae) and insights on the origin of gold-of-pleasure (*Camelina sativa*). Mol Phylogenet Evol. 127:834–842. 10.1016/j.ympev.2018.06.031. 29933039

[kiag052-B11] Brock JR, Scott T, Lee AY, Mosyakin SL, Olsen KM. 2020. Interactions between genetics and environment shape Camelina seed oil composition. BMC Plant Biol. 20:423. 10.1186/s12870-020-02641-8. 32928104 PMC7490867

[kiag052-B12] Byfield GE, Upchurch RG. 2007. Effect of temperature on Delta-0 stearoyl-ACP and microsomal Omega-6 desaturase gene expression and fatty acid content in developing soybean seeds. Crop Sci. 47:1698–1704. 10.2135/cropsci2006.04.0213.

[kiag052-B13] Čanak P et al 2020. Is drought stress tolerance affected by genotypes and seed size in the emerging oilseed crop Camelina? Agronomy. 10:1856. 10.3390/agronomy10121856.

[kiag052-B14] Carey LM et al 2020. High flux through the oxidative pentose phosphate pathway lowers efficiency in developing Camelina seeds. Plant Physiol. 182:493–506. 10.1104/pp.19.00740. 31699846 PMC6945844

[kiag052-B15] Chaudhary R et al 2020. Assessing diversity in the Camelina genus provides insights into the genome structure of *Camelina sativa*. G3 (Bethesda). 10:1297–1308. 10.1534/g3.119.400957. 32046969 PMC7144077

[kiag052-B16] Choudhury FK, Rivero RM, Blumwald E, Mittler R. 2017. Reactive oxygen species, abiotic stress and stress combination. Plant J. 90:856–867. 10.1111/tpj.13299. 27801967

[kiag052-B17] Decker S, Craine W, Paulitz T, Chen CC, Lu CF. 2025. Genomic analysis of the natural variation of fatty acid composition in seed oils of *Camelina sativa*. Biology (Basel). 14:1199. 10.3390/biology14091199. 41007343 PMC12467081

[kiag052-B18] Decros G et al 2019. Get the balance right: ROS homeostasis and redox signalling in fruit. Front Plant Sci. 10:1091. 10.3389/fpls.2019.01091. 31620143 PMC6760520

[kiag052-B19] Djoumbou Feunang Y et al 2016. ClassyFire: automated chemical classification with a comprehensive, computable taxonomy. J Cheminform. 8:61. 10.1186/s13321-016-0174-y. 27867422 PMC5096306

[kiag052-B20] Dussarrat T et al 2022. Predictive metabolomics of multiple Atacama plant species unveils a core set of generic metabolites for extreme climate resilience. New Phytol. 234:1614–1628. 10.1111/nph.18095. 35288949 PMC9324839

[kiag052-B21] Fernandez O et al 2016. Fortune telling: metabolic markers of plant performance. Metabolomics. 12:158. 10.1007/s11306-016-1099-1. 27729832 PMC5025497

[kiag052-B22] Fernandez O et al 2021. Plant metabolomics and breeding. Adv Bot Res. 98:207–235. 10.1016/bs.abr.2020.09.020.

[kiag052-B23] Gao L, Caldwell CD, Jiang Y. 2018. Photosynthesis and growth of Camelina and canola in response to water deficit and applied nitrogen. Crop Sci. 58:393–401. 10.2135/cropsci2017.07.0406.

[kiag052-B24] Gesch RW, Cermak SC. 2011. Sowing date and tillage effects on fall-seeded camelina in the northern corn belt. Agron J. 103:980–987. 10.2134/agronj2010.0485.

[kiag052-B25] Gishini MFS, Kachroo P, Hildebrand D. 2025. Fatty acid desaturase 3-mediated α-linolenic acid biosynthesis in plants. Plant Physiol. 197:kiaf012. 10.1093/plphys/kiaf012. 39823389

[kiag052-B26] Großkinsky D et al 2023. The potential of integrative phenomics to harness underutilized crops for improving stress resilience. Front Plant Sci. 14:1216337. 10.3389/fpls.2023.1216337. 37409292 PMC10318926

[kiag052-B27] Heinemann B, Hildebrandt TM. 2021. The role of amino acid metabolism in signalling and metabolic adaptation to stress-induced energy deficiency in plants. J Exp Bot. 72:4634–4645. 10.1093/jxb/erab182. 33993299

[kiag052-B28] Higashi Y, Sato K. 2019. Lipidomic studies of membrane glycerolipids in plants leaves under heat stress. Prog Lipid Res. 75:100990. 10.1016/j.plipres.2019.100990. 31442527

[kiag052-B29] Hildebrandt TM . 2018. Synthesis versus degradation: directions of amino acid metabolism during Arabidopsis abiotic stress response. Plant Mol Biol. 98:121–135. 10.1007/s11103-018-0767-0. 30143990

[kiag052-B30] Hotton SK et al 2020. Phenotypic examination of *Camelina sativa* (L.) Crantz accessions from the USDA-ARS national genetics resource program. Plants. 9:642. 10.3390/plants9050642. 32438618 PMC7286027

[kiag052-B31] Hummel I et al 2010. Arabidopsis plants acclimate to water deficit at low cost through changes of carbon usage: an integrated perspective using growth, metabolite, enzyme, and gene expression analysis. Plant Physiol. 154:357–372. 10.1104/pp.110.157008. 20631317 PMC2938159

[kiag052-B32] Jensen CR et al 1996. Seed glucosinolate, oil and protein contents of field-grown rape (*Brassica napus* L.) affected by soil drying and evaporative demand. Field Crop Res. 47:93–105. 10.1016/0378-4290(96)00026-3.

[kiag052-B33] Kagale S et al 2014. The emerging biofuel crop *Camelina sativa* retains a highly undifferentiated hexaploid genome structure. Nat Commun. 5:3706. 10.1038/ncomms4706. 24759634 PMC4015329

[kiag052-B34] King K, Li H, Kang J, Lu C. 2019. Mapping quantitative trait loci for seed traits in *Camelina sativa*. Theor Appl Genet. 132:2567–2577. 10.1007/s00122-019-03371-8. 31177293

[kiag052-B35] Kumar V et al 2025. Deciphering the role of monoacylglycerol lipases under abiotic stress and lipid metabolism in soybean (*Glycine max* L.). Plant Biotechnol J. 23:4318–4335. 10.1111/pbi.70088. 40596782 PMC12483956

[kiag052-B36] Lamari N et al 2018. Metabotyping of 30 maize hybrids under early-sowing conditions reveals potential marker-metabolites for breeding. Metabolomics. 14:132. 10.1007/s11306-018-1427-8. 30830438 PMC6208756

[kiag052-B37] Lesk C et al 2021. Stronger temperature–moisture couplings exacerbate the impact of climate warming on global crop yields. Nat Food. 2:683–691. 10.1038/s43016-021-00341-6. 37117467

[kiag052-B38] Lesk C, Rowhani P, Ramankutty N. 2016. Influence of extreme weather disasters on global crop production. Nature. 529:84–87. 10.1038/nature16467. 26738594

[kiag052-B39] Li H et al 2021. Genetic dissection of natural variation in oilseed traits of camelina by whole-genome resequencing and QTL mapping. Plant Genome. 14:e20110. 10.1002/tpg2.20110. 34106529 PMC12806864

[kiag052-B40] Luna E et al 2020. Metabolomics to exploit the primed immune system of tomato fruit. Metabolites. 10:96. 10.3390/metabo10030096. 32155921 PMC7143431

[kiag052-B41] Luo Z et al 2019. Genetic diversity and population structure of a *Camelina sativa* spring panel. Front Plant Sci. 10:184. 10.3389/fpls.2019.00184. 30842785 PMC6391347

[kiag052-B42] Mandáková T, Lysak MA. 2022. The identification of the missing maternal genome of the allohexaploid camelina (*Camelina sativa*). Plant J. 112:622–629. 10.1111/tpj.15931. 35916590

[kiag052-B43] Mandáková T, Pouch M, Brock JR, Al-Shehbaz IA, Lysak MA. 2019. Origin and evolution of diploid and allopolyploid Camelina genomes were accompanied by chromosome shattering. Plant Cell. 31:2596–2612. 10.1105/tpc.19.00366. 31451448 PMC6881126

[kiag052-B44] Martinelli T, Galasso I. 2011. Phenological growth stages of *Camelina sativa* according to the extended BBCH scale. Annals App Biol. 158:87–94. 10.1111/j.1744-7348.2010.00444.x.

[kiag052-B45] Mishra S, Spaccarotella K, Gido J, Samanta I, Chowdhary G. 2023. Effects of heat stress in plant-nutrient relations: an update on nutrient uptake, transport, and assimilation. Int J Mol Sci. 24:15670. 10.3390/ijms242115670. 37958654 PMC10649217

[kiag052-B46] Nadakuduti SS et al 2023. Heat stress during seed development leads to impaired physiological function and plasticity in seed oil accumulation in *Camelina sativa*. Front Plant Sci. 14:1284573. 10.3389/fpls.2023.1284573. 38078110 PMC10704172

[kiag052-B47] Pang Z et al 2021. MetaboAnalyst 5.0: narrowing the gap between raw spectra and functional insights. Nucleic Acids Res. 49:W388–W396. 10.1093/nar/gkab382. 34019663 PMC8265181

[kiag052-B48] Poplin R et al 2018 Jul 24. Scaling accurate genetic variant discovery to tens of thousands of samples. bioRxiv 201178. 10.1101/201178, preprint; not peer reviewed.

[kiag052-B49] Poucet T et al 2021. Ammonium supply induces differential metabolic adaptive responses in tomato according to leaf phenological stage. J Exp Bot. 72:3185–3199. 10.1093/jxb/erab057. 33578414

[kiag052-B50] Prado K et al 2025. Building climate-resilient crops: genetic, environmental, and technological strategies for heat and drought stress tolerance. J Exp Bot. 76:4395–4413. 10.1093/jxb/eraf111. 40062368

[kiag052-B51] Purcell S et al 2007. PLINK: a tool set for whole-genome association and population-based linkage analyses. Am J Hum Genet. 81:559–575. 10.1086/519795. 17701901 PMC1950838

[kiag052-B52] Rankenberg T et al 2021. Age-dependent abiotic stress resilience in plants. Trends Plant Sci. 26:692–705. 10.1016/j.tplants.2020.12.016. 33509699

[kiag052-B53] Ray DK, Gerber JS, MacDonald GK, West PC. 2015. Climate variation explains a third of global crop yield variability. Nat Commun. 6:5989. 10.1038/ncomms6989. 25609225 PMC4354156

[kiag052-B54] Robinson D . 2001. δ^15^N as an integrator on the nitrogen cycle. Trends Ecol Evol. 16:153–162. 10.1016/S0169-5347(00)02098-X. 11179580

[kiag052-B55] Secchi MA et al 2023. Effects of heat and drought on canola (*Brassica napus* L.) yield, oil and protein: a meta-analysis. Field Crops Res. 293:108848. 10.1016/j.fcr.2023.108848.

[kiag052-B56] Sharma P et al 2023. Drought and heat stress mediated activation of lipid signaling in plants: a critical review. Front Plant Sci. 14:1216835. 10.3389/fpls.2023.1216835. 37636093 PMC10450635

[kiag052-B57] Smith BE, Kemmer S, Decker S, Lu C. 2024. Quantitative trait locus (QTL) mapping and transcriptome profiling identify QTLs and candidate genes associated with heat stress response during reproductive development in *Camelina sativa*. Food Energy Secur. 13:e531. 10.1002/fes3.531.

[kiag052-B58] Smith BE, Lu C. 2024. Heat stress during reproductive stages reduces camelina seed productivity and changes seed composition. Heliyon. 10:e26678. 10.1016/j.heliyon.2024.e26678. 38434085 PMC10907518

[kiag052-B59] Stasnik P, Großkinsky DK, Jonak C. 2022. Physiological and phenotypic characterization of diverse *Camelina sativa* lines in response to waterlogging. Plant Physiol Biochem. 183:120–127. 10.1016/j.plaphy.2022.05.007. 35580367

[kiag052-B60] Stasnik P, Vollmann J, Großkinsky DK, Jonak C. 2024. Leaf carbohydrate metabolic enzyme activities are associated with salt tolerance and yield stability in the climate-resilient crop *Camelina sativa*. Plant Stress. 14:100629. 10.1016/j.stress.2024.100629.

[kiag052-B61] Thalman M, Santelia D. 2017. Starch as a determinant of plant fitness under abiotic stress. New Phytol. 214:943–951. 10.1111/nph.14491. 28277621

[kiag052-B62] Upchurch RG . 2008. Fatty acid unsaturation, mobilization and regulation in the response of plants to stress. Biotechnol Lett. 30:967–977. 10.1007/s10529-008-9639-z. 18227974

[kiag052-B63] Vollmann J, Moritz T, Kargl C, Baumgartner S, Wagentristl H. 2007. Agronomic evaluation of camelina genotypes selected for seed quality characteristics. Ind Crop Prod. 26:270–277. 10.1016/j.indcrop.2007.03.017.

[kiag052-B64] Xu Y et al 2025. Metabolic marker-assisted genomic prediction improves hybrid breeding. Plant Commun. 6:101199. 10.1016/j.xplc.2024.101199. 39614617 PMC11956108

[kiag052-B65] Yu E et al 2014. Identification of heat responsive genes in *Brassica napus* siliques at the seed-filling stage through transcriptional profiling. PLoS One. 9:e101914. 10.1371/journal.pone.0101914. 25013950 PMC4094393

[kiag052-B66] Zandalinas SI, Balfagón D, Gómez-Cadenas A, Mittler R. 2022. Plant responses to climate change: metabolic changes under combined abiotic stresses. J Exp Bot. 73:3339–3354. 10.1093/jxb/erac073. 35192700

[kiag052-B67] Zanetti F et al 2021. Camelina, an ancient oilseed crop actively contributing to the rural renaissance in Europe. A review. Agron Sustain Dev. 41:2. 10.1007/s13593-020-00663-y.

[kiag052-B68] Zubr J . 1997. Oil-seed crop: *Camelina sativa*. Ind Crop Prod. 6:113–119. 10.1016/S0926-6690(96)00203-8.

