# Supplementary material for: Assessment of phenotypic trait plasticity in the oilseed Camelina sativa using integrated early stage abiotic stress and field studies
Source: Plant Physiol. 2026 Feb 11;200(3):kiag052. doi: 10.1093/plphys/kiag052 (PMC13017834; doi:10.1093/plphys/kiag052)
Supplement: kiag052_Supplementary_Data [file kiag052_supplementary_data.pdf]

Supplementary Figures

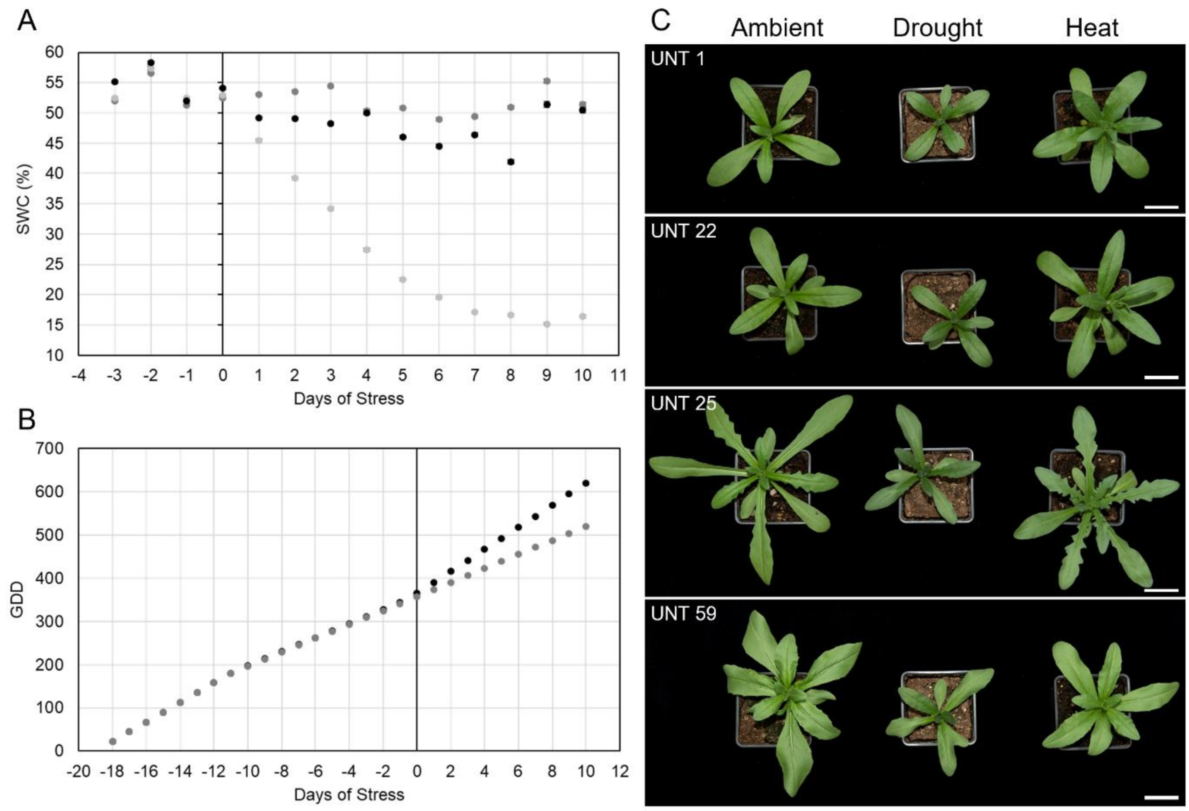

Supplementary Figure S1. Camelina diversity panel early-stage abiotic stress treatments. (A) Soil water content (SWC) per days of stress for ambient (●), heat (●), and drought (●) stresses. (B) Growing degree days (GDD) per days of stress for ambient (●) and heat (●) glasshouses. (C) Illustrative examples of the camelina diversity panel lines responding to abiotic stress in early-stage drought and heat stress. Plants were grown at ambient temperature and well-watered (Ambient, 20/18°C day/night, 50% soil water content, SWC); ambient temperature and 20% SWC (Drought); and at 32/25°C day/night and 50% SWC (Heat). Scale bar = 5 cm.

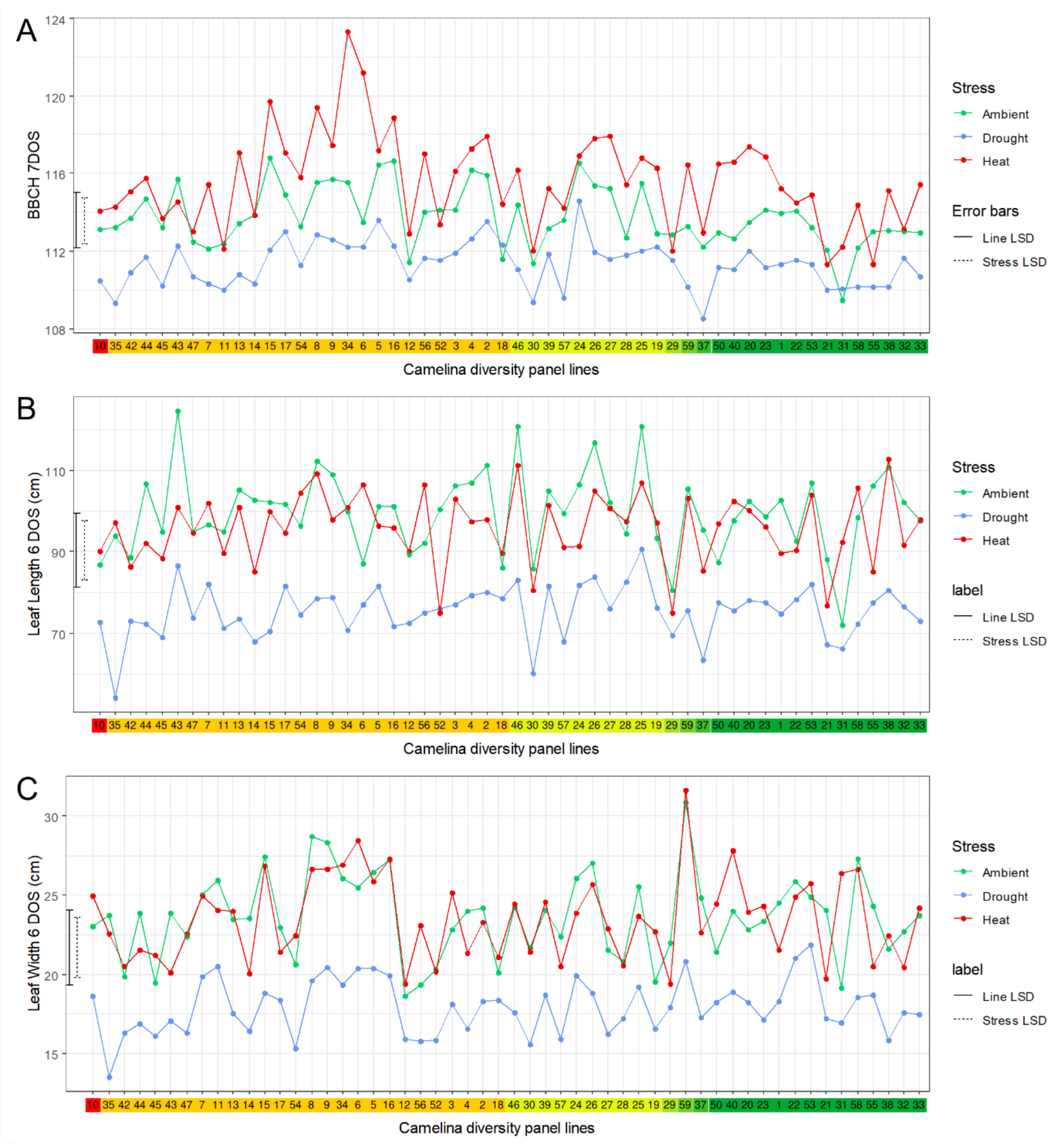

Supplementary Figure S2. Phenotypic responses of the camelina diversity panel to early-stage abiotic stress treatments. (A) Plant development (BBCH scale) scored at seven days after stress (7DOS). (B) Leaf length (cm) measured at six days of stress (6DOS). (C) Leaf width (mm) measured at six days of stress (6DOS). Ambient (green), heat (red) and drought (blue) stresses. The solid black line illustrates least significant difference (LSD) between lines, and hatched black line indicates the LSD between stresses. Interactions between Stress and Line were non-significant for both leaf width and leaf length. The diversity panel lines (x axis) were plotted and colour coded based on the distributions determined by hierarchical clustering and admixture population genetics analysis shown in Fig. 1B.

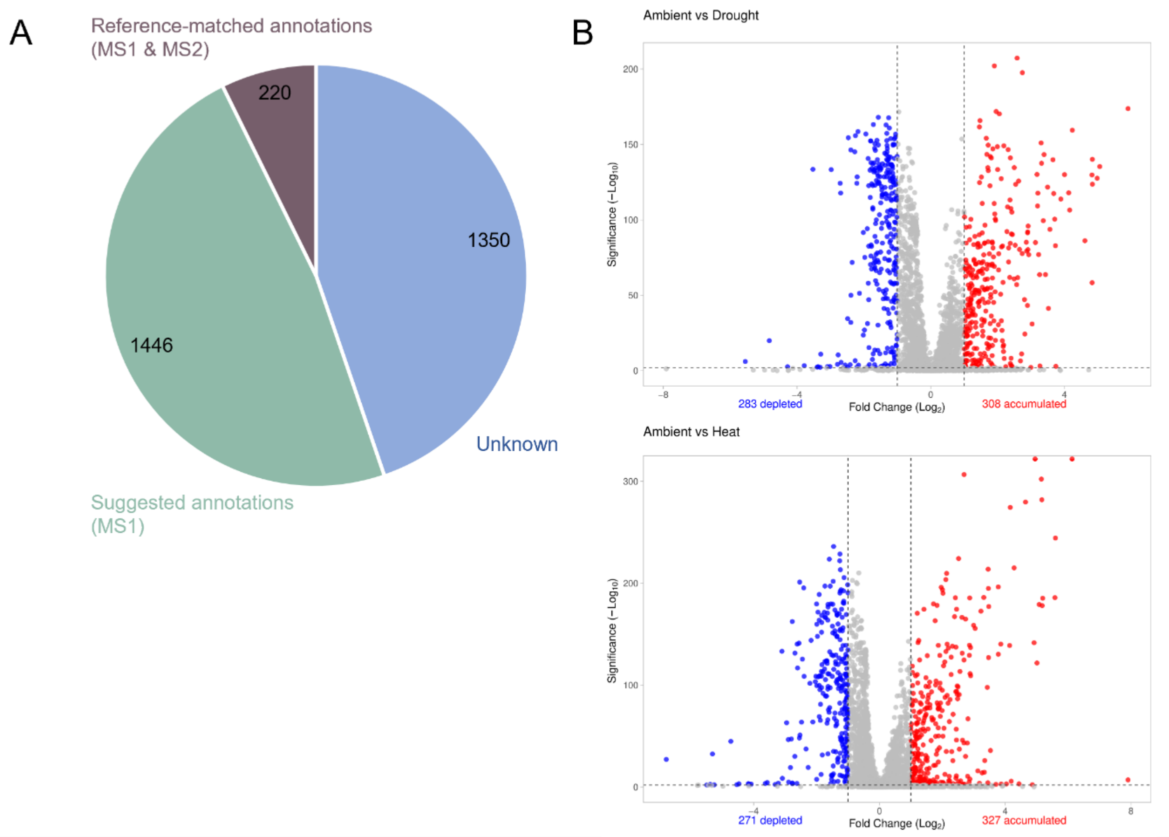

Supplementary Figure S3. Untargeted metabolomics of the camelina diversity panel in response to early-stage abiotic stress. (A) Distribution of the 3016 metabolomic features according to their annotation level by MS-DIAL processing. (B) Volcano plots (Fold change, FC > 2; $P < 0.01$, FDR corrected) of metabolic markers that were accumulated (red) or depleted (blue) in response to drought (top panel) or heat stress (bottom panel).

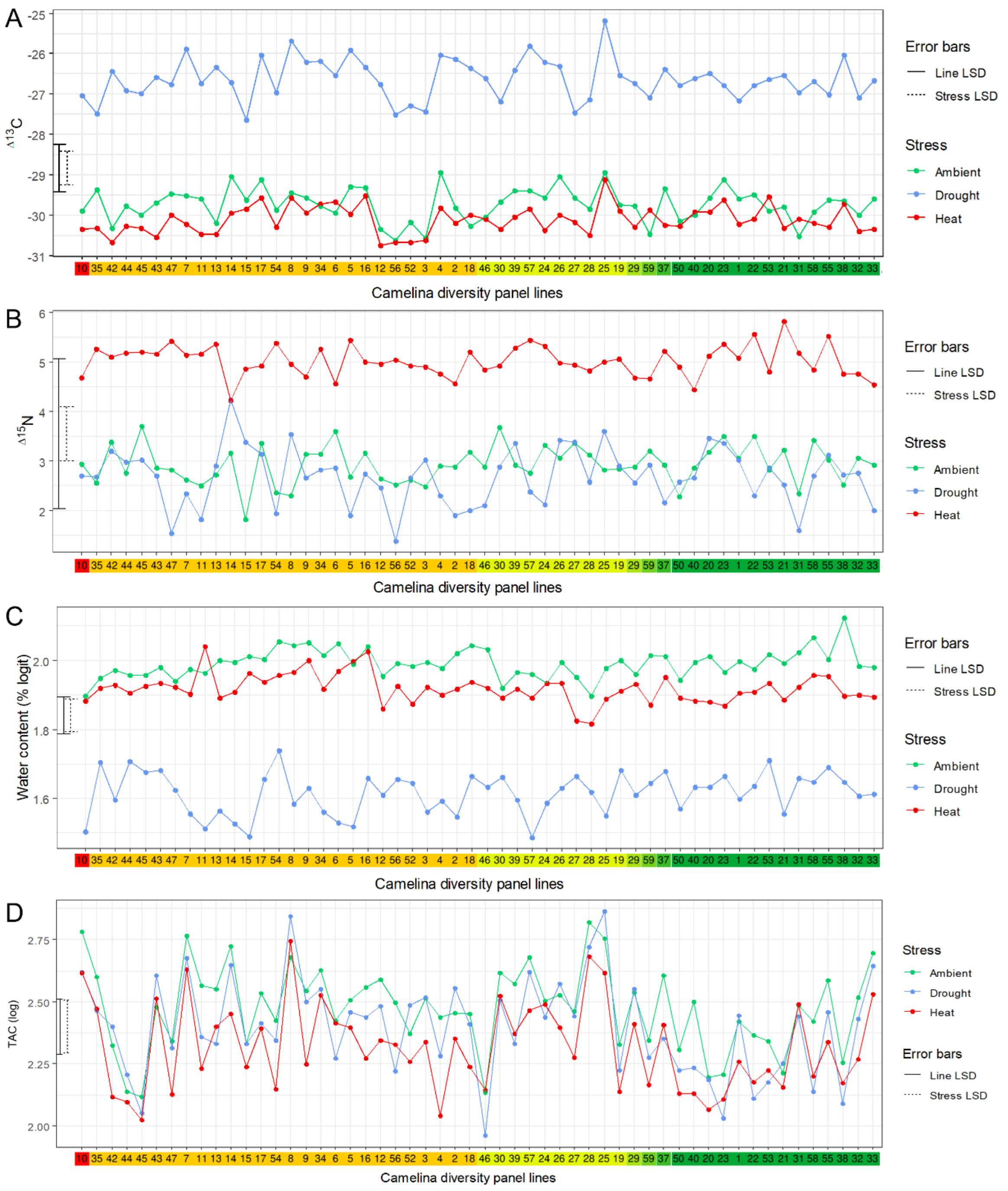

Supplementary Figure S4. Leaf stable isotope analysis, plant water content and total oxidant capacity for the camelina diversity panel in response to early-stage abiotic stress. (A) $\Delta^{13}C$, (B) $\Delta^{15}N$ leaf stable isotope analysis; (C) percentage plant water content (PWC, % logit); and (D) total antioxidant capacity (TAC, U/mg Protein, log transformed data). Ambient (green), drought (blue), heat (red). The diversity panel lines (x axis) were plotted and colour coded based on the distributions determined hierarchical clustering and admixture population genetics analysis in Fig 1. Error bars illustrate least significant difference (LSD) between lines (solid black) and between stresses (dashed black). Interaction between stress and line was non-significant for $\Delta^{13}C$, $\Delta^{15}N$, and TAC.

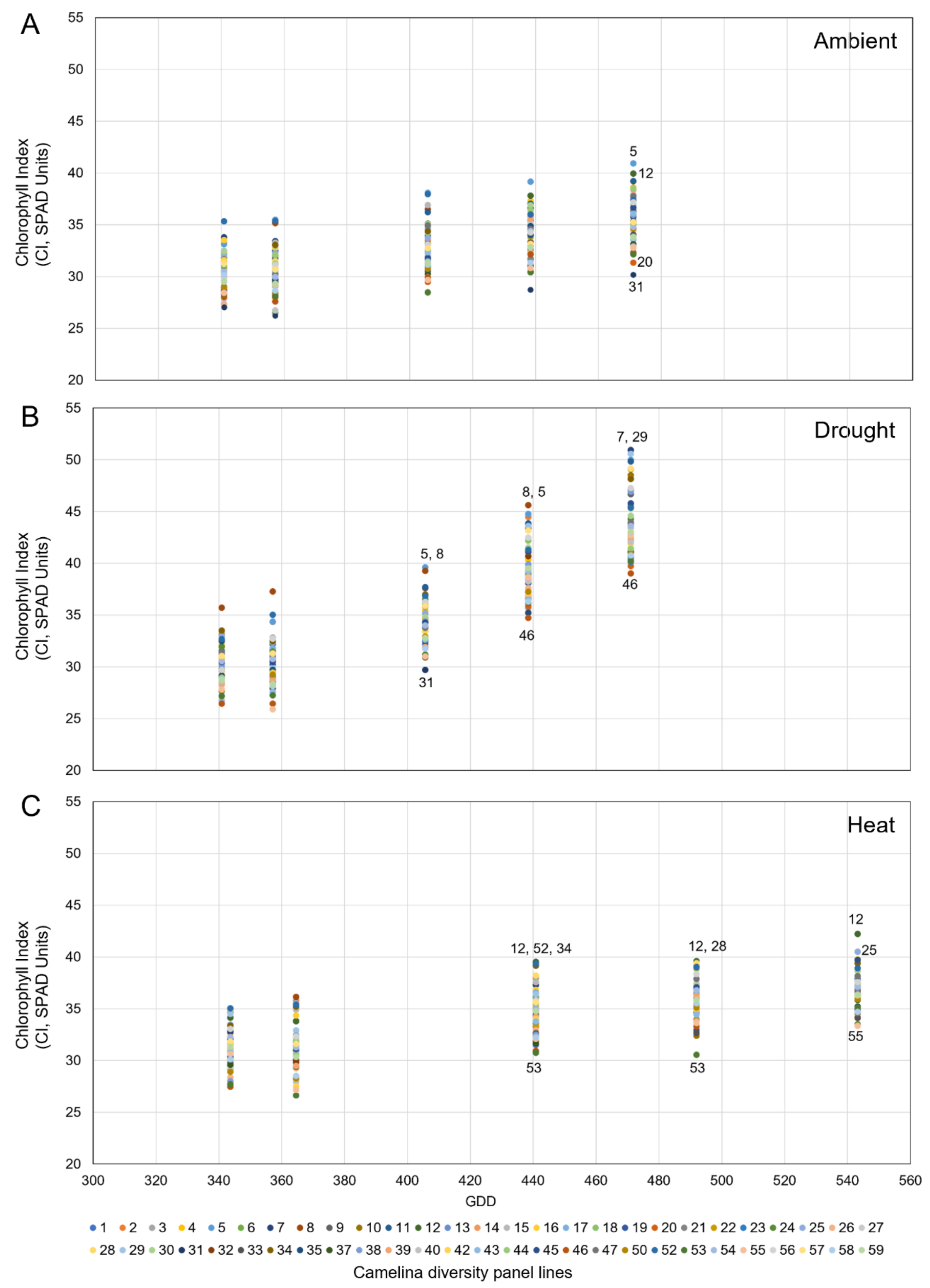

Supplementary Figure S5. Camelina diversity panel leaf chlorophyll content determined by SPAD-502 Plus. Chlorophyll content is expressed per accumulated growing degree days (GDD) for ambient (A), drought (B), and heat (C). Values represent a mean of five replicate measurements per line and treatment. Line numbers shown in graph to represent top and bottom performers in each treatment applied.

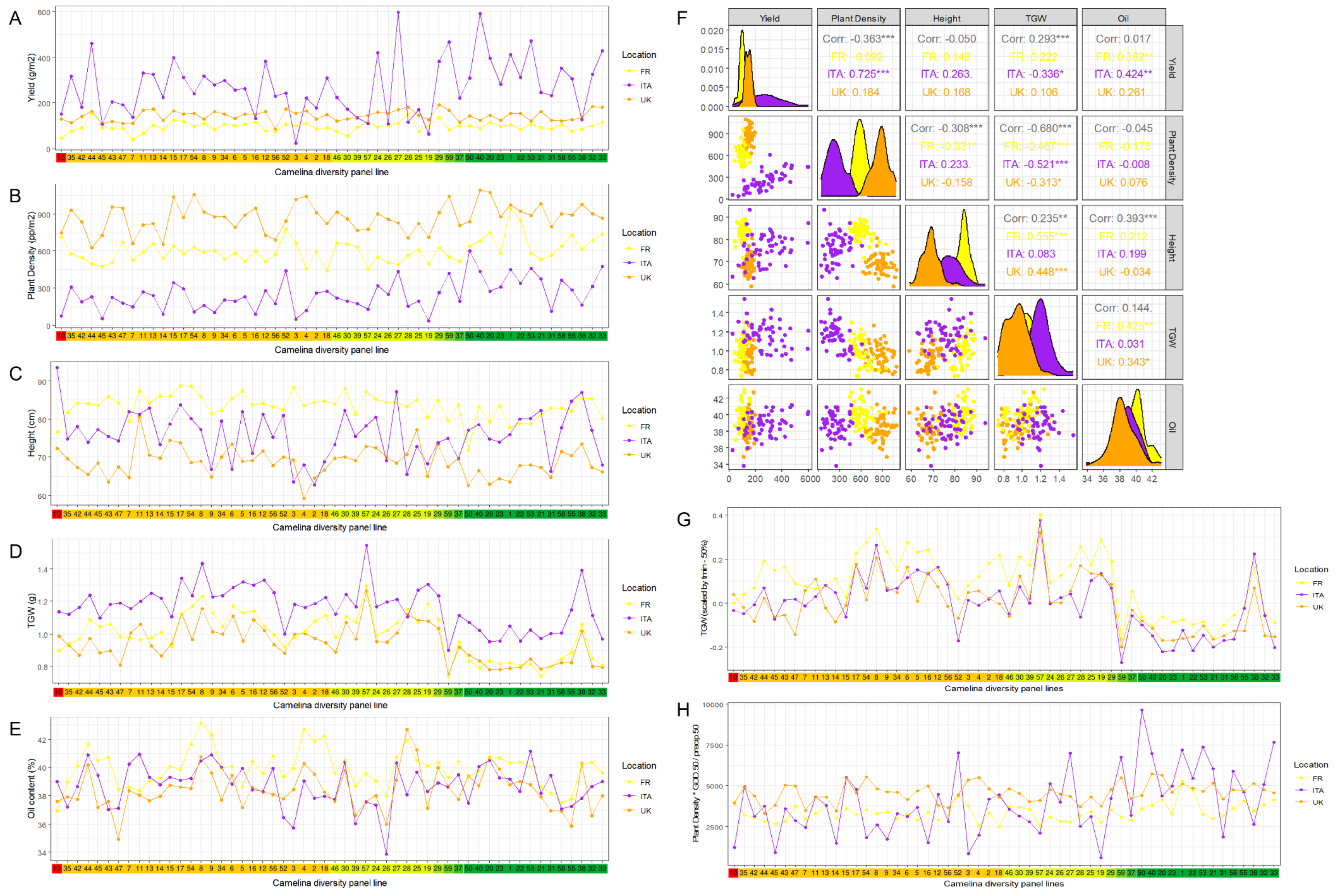
A
Yield (g/m2)
600
400
200
0
10 35 42 44 45 43 47 7 11 13 14 15 17 54 8 9 34 6 5 16 12 56 52 3 4 2 18 46 30 39 57 24 26 27 28 25 19 29 59 37 50 40 20 23 1 22 53 21 31 58 55 38 32 33
Camelina diversity panel line
Location
FR
ITA
UK
B
Plant Density (pp/m2)
900
600
300
0
10 35 42 44 45 43 47 7 11 13 14 15 17 54 8 9 34 6 5 16 12 56 52 3 4 2 18 46 30 39 57 24 26 27 28 25 19 29 59 37 50 40 20 23 1 22 53 21 31 58 55 38 32 33
Camelina diversity panel line
Location
FR
ITA
UK
C
Height (cm)
90
80
70
60
10 35 42 44 45 43 47 7 11 13 14 15 17 54 8 9 34 6 5 16 12 56 52 3 4 2 18 46 30 39 57 24 26 27 28 25 19 29 59 37 50 40 20 23 1 22 53 21 31 58 55 38 32 33
Camelina diversity panel line
Location
FR
ITA
UK
D
TGW (g)
1.4
1.2
1.0
0.8
10 35 42 44 45 43 47 7 11 13 14 15 17 54 8 9 34 6 5 16 12 56 52 3 4 2 18 46 30 39 57 24 26 27 28 25 19 29 59 37 50 40 20 23 1 22 53 21 31 58 55 38 32 33
Camelina diversity panel line
Location
FR
ITA
UK
E
Oil content (%)
42
40
38
36
34
10 35 42 44 45 43 47 7 11 13 14 15 17 54 8 9 34 6 5 16 12 56 52 3 4 2 18 46 30 39 57 24 26 27 28 25 19 29 59 37 50 40 20 23 1 22 53 21 31 58 55 38 32 33
Camelina diversity panel line
Location
FR
ITA
UK
F
Yield
Plant Density
Height
TGW
Oil
Corr: -0.363***
FR: -0.092
ITA: 0.725***
UK: 0.184
Corr: -0.050
FR: 0.148
ITA: 0.263.
UK: 0.168
Corr: 0.293***
FR: 0.222
ITA: -0.336*
UK: 0.106
Corr: 0.017
FR: 0.382**
ITA: 0.424**
UK: 0.261.
Corr: -0.308***
FR: -0.331*
ITA: 0.233.
UK: -0.158
Corr: -0.680***
FR: -0.467***
ITA: -0.521***
UK: -0.313*
Corr: -0.045
FR: -0.175
ITA: -0.008
UK: 0.076
Corr: 0.235**
FR: 0.555***
ITA: 0.083
UK: 0.448***
Corr: 0.393***
FR: 0.212
ITA: 0.199
UK: -0.034
Corr: 0.144.
FR: 0.425**
ITA: 0.031
UK: 0.343*
0.020
0.015
0.010
0.005
0.000
900
600
300
0
90
80
70
60
1.4
1.2
1.0
0.8
42
40
38
36
34
0 200 400 6000 300 600 900 60 70 80 90 0.8 1.0 1.2 1.4 34 36 38 40 42
G
TGW (scaled by tmin - 50%)
0.4
0.2
0.0
-0.2
10 35 42 44 45 43 47 7 11 13 14 15 17 54 8 9 34 6 5 16 12 56 52 3 4 2 18 46 30 39 57 24 26 27 28 25 19 29 59 37 50 40 20 23 1 22 53 21 31 58 55 38 32 33
Camelina diversity panel lines
Location
FR
ITA
UK
H
Plant Density * GDD 50 / precip 50
10000
7500
5000
2500
10 35 42 44 45 43 47 7 11 13 14 15 17 54 8 9 34 6 5 16 12 56 52 3 4 2 18 46 30 39 57 24 26 27 28 25 19 29 59 37 50 40 20 23 1 22 53 21 31 58 55 38 32 33
Camelina diversity panel lines
Location
FR
ITA
UK

Supplementary Figure S6. Field trial performance of the camelina diversity panel grown in multi-location field trials. (A) yield (g/m^2), (B) plant density ($plants/m^2$), (C) plant height (cm), (D) thousand grain weight (TGW, g), and (E) seed oil content (% of weight). (F) Pairwise scatterplots of country specific predicted means for each measured trait and line. Correlation values (Corr) show the Pearson correlation coefficient between traits for all countries. (G) TGW (g) adjusted by the scaled minimum temperature (T_{min}, °C) at 50% flowering. (H) Plant density rescaled by a factor of GDD (at 50% flowering) / precipitation (at 50% flowering); GDD, growing degree days. UK (orange), France (FR, yellow), and Italy (ITA, purple). Points are the mean over the field replicates. For all panels, except (F), the diversity panel lines (x axis) were plotted and coloured based on the distributions determined by hierarchical clustering and admixture population genetics analysis in Fig. 1B.

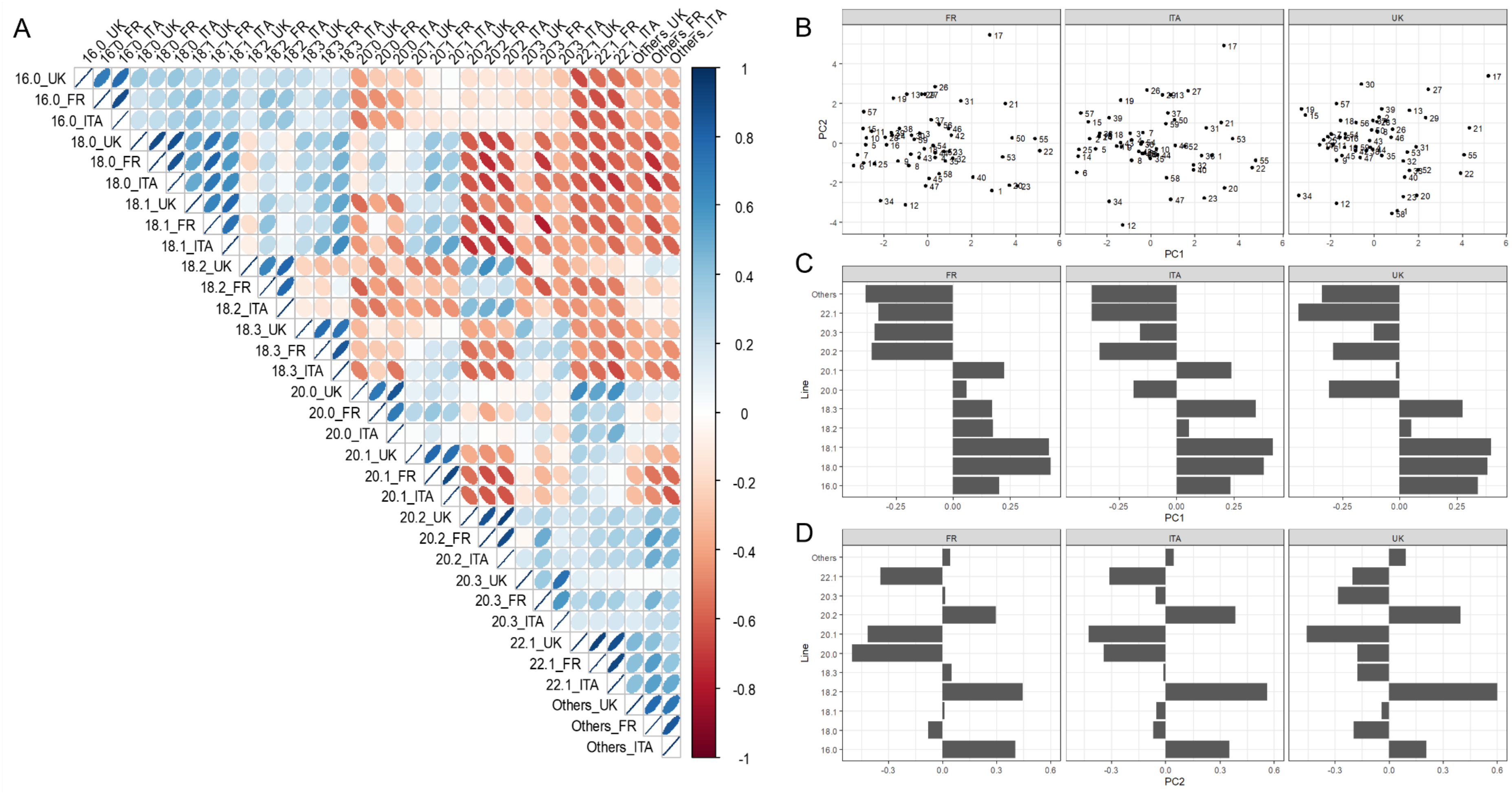

Supplementary Figure S7. Analysis of seed fatty acids of the camelina diversity panel grown in multi-location field trials. (A) Pearson correlation coefficients are depicted between the predicted genotypic means of each fatty acid (FA; number of carbons.desaturations) within each country France (FR), Italy (ITA) and UK, and calculated on the centred log ratio scale. 54 data points were used to calculate each correlation coefficient. The narrower and darker the ellipse, the stronger the correlation with blue values indicating a positive correlation and red

indicating a negative correlation. (B) Principal components analysis (PCA) on the predicted genotypic seed FA means for each individual line, The contributions of PC1 and PC2 are shown in C and D respectively for each location and FA. Note for France PC1 and 2 explain 43% and 23%; Italy PC 1 and PC 2 explain 41% and 22%, and for the UK PC 1 and PC 2 explain 35% and 21% of the total variation.

Supplementary Tables

Supplementary Table S1. Name and origin of the camelina diversity panel.

Experimental Code	Name1	Name2	Name3	Origin	Geographical origin	Provider
UNT1	UNT1			CCE breeding program	Spain	CCE
UNT2	UNT2			CCE breeding program	Spain	CCE
UNT3	UNT3			CCE breeding program	Spain	CCE
UNT4	UNT4			CCE breeding program	Spain	CCE
UNT5	UNT5			CCE breeding program	Spain	CCE
UNT6	UNT6			CCE breeding program	Spain	CCE
UNT7	UNT7			CCE breeding program	Spain	CCE
UNT8	UNT8			CCE breeding program	Spain	CCE
UNT9	UNT9			CCE breeding program	Spain	CCE
UNT10	UNT10			CCE breeding program	Spain	CCE
UNT11	UNT11			CCE breeding program	Spain	CCE
UNT12	UNT12			CCE breeding program	Spain	CCE
UNT13	UNT13			CCE breeding program	Spain	CCE
UNT14	UNT14			CCE breeding program	Spain	CCE
UNT15	UNT15			CCE breeding program	Spain	CCE
UNT16	UNT16			CCE breeding program	Spain	CCE
UNT17	UNT17			CCE breeding program	Spain	CCE
UNT18	Celine			Public accession	France	INRAe
UNT19	CN101981			Public accession	Poland	INRAe
UNT20	CN101985			Public accession	unknown	INRAe
UNT21	CN101980	Przybrodzka	PI 311736	Public accession	Poland	INRAe
UNT22	CN101982			Public accession	Former Yugoslavia	INRAe
UNT23	CN101986	Giessen N°3	PI 650148	Public accession	Germany	INRAe
UNT24	CN111335	Chulymskij		Public accession	Russia	INRAe
UNT25	CN111672	PI258366		Public accession	Russia	INRAe
UNT26	CN111330	VIR-4027		Public accession	Russia	INRAe
UNT27	CN30475	Voronezskij 349	Cs110 CAM 174	Public accession	Russia	INRAe
UNT28	CN30476	Voronezskij 339		Public accession	Russia	INRAe
UNT29	CN30478	Omskij Mestnyj	Cs006, CAM 25	Public accession	Russia	INRAe
UNT30	CN30479	Irkuskiij Mestnyj		Public accession	Russia	INRAe
UNT31	CN101987	Giessen N°4	PI 650149, Cs218	Public accession	Germany	INRAe
UNT32	CN101988	Hoga	PI 650150, Cs035, CAM 67	Public accession	Denmark	INRAe

(Continued on next page)

Experimental Code	Name1	Name2	Name3	Origin	Geographical origin	Provider
UNT33	CN101989			Public accession	Unknown	INRAe
UNT34	Calena	Cs076	CAM 134	Public accession	Austria	AIT
UNT35	Gomholka			Public accession	Hungary	AIT
UNT37	Ya-ma-ji			Public accession	China	AIT
UNT38	Kirgizskij	Cs028	CAM 57	Public accession	Kyrgyzstan	AIT
UNT39	Irkutskij local			Public accession	Russia	AIT
UNT40	Korneuburg			Public accession	Austria	AIT
UNT42	CA13X_15_21			BOKU accession	Austria	UNIBO
UNT43	CJ6X_78			BOKU accession	Austria	UNIBO
UNT44	CK1X_129			BOKU accession	Austria	UNIBO
UNT45	BGRC51558			Public accession	Germany	UNIBO
UNT46	CUOO5	Omskij		Public accession	Russia	UNIBO
UNT47	CJ13X-115			Public accession	Austria	UNIBO
UNT50	Cs083	CAM 147	STAMM 09X13	Public accession	Germany	INRAe
UNT52	Cs092	CAM 156	STAMM 09X15	Public accession	Germany	INRAe
UNT53	Cs095	CAM 159	STAMM 13X15	Public accession	Germany	INRAe
UNT54	Cs101	CAM 165	STAMM 04X05	Public accession	Germany	INRAe
UNT55	Cs103	CAM 167	STAMM 06X14C	Public accession	Germany	INRAe
UNT56	Cs151	CAM 232	PRFGL. 85	Public accession	Germany	INRAe
UNT57	Cs193	CAM 278	STAMM 02X11B	Public accession	Germany	INRAe
UNT58	Cs205	PI 597833	163-2073-72	Public accession	Denmark	INRAe
UNT59	Cs233	PI 650164	CSS-CAM38	Public accession	Austria	INRAe

CCE, Camelina Company España; INRAe, AgroParisTech Université Paris-Saclay; AIT, Austrian Institute of Technology; UNIBO, Università di Bologna; BOKU, University of Natural Resources and Life Sciences, Vienna.

Supplementary Table S2. Statistical analysis of measured parameters for the camelina panel response to early-stage abiotic stress treatments. Linear mixed models were fitted using REML (restricted maximum likelihood) to each variable with random structure accounting for the blocking imposed in the design (Glasshouse/Tray/Pot) and treatment structure Line * Treatment (TRT). Treatment indicates abiotic stress (heat or drought). Approximate F-tests were calculated using the Satterthwaite approximation. All models were fitted in R using lme4 and lmerTest packages. NumDF, numerator degrees of freedom; denDF, denominator degrees of freedom; interactions denoted by a ":"; significance codes are '***' 0.001; '**' 0.05; '*' 0.1.

A. Developmental stage 1 (BBCH scale) following seven days of stress

Source of Variation	Sum Sq	Mean Sq	NumDF	DenDF	F value	Pr(>F)	Sig.
LINE	1671.65	31.540	53	598.73	9.6448	2.2e-16	***
TRT	84.59	42.296	2	5.98	12.9339	0.006735	**
LINE:TRT	663.65	6.261	106	596.37	1.9145	1.2e-6	***

B. Leaf length after six days of stress

Source of Variation	Sum Sq	Mean Sq	NumDF	DenDF	F value	Pr(>F)	Sig.
LINE	35714	673.85	53	603.88	5.1621	2.2e-16	***
TRT	3003	1501.46	2	5.98	11.5019	8.936e-3	**
LINE:TRT	16855	159	106	600.06	1.2181	0.082559	

C. Leaf width after six days of stress

Source of Variation	Sum Sq	Mean Sq	NumDF	DenDF	F value	Pr(>F)	Sig.
LINE	3202.2	60.420	53	600.76	6.9952	2e-16	***
TRT	183.5	91.741	2	5.98	10.6214	0.01076	*
LINE:TRT	990.1	9.34	106	598.15	1.0814	0.28637	

D. Total leaf fatty acids

Source of Variation	Sum Sq	Mean Sq	NumDF	DenDF	F value	Pr(>F)	Sig.
LINE	4.7202	0.0891	53	583.28	0.6648	0.9673	
TRT	16.3743	8.1871	2	5.96	61.1106	1.069e-4	***
LINE:TRT	17.5752	0.1658	106	581.39	1.2376	0.0674	

E. Leaf relative polyunsaturated fatty acid content (PUFA index)

Source of Variation	Sum Sq	Mean Sq	NumDF	DenDF	F value	Pr(>F)	Sig.
LINE	9.7462	0.18389	53	583.78	1.1436	0.232964	
TRT	5.0439	2.52194	2	5.98	15.6835	0.004191	**

LINE:TRT	14.2891	0.1348	106	581.4	0.8383	0.868914	

F. Leaf C18:3 fatty acid content

Source of Variation	Sum Sq	Mean Sq	NumDF	DenDF	F value	Pr(>F)	Sig.
LINE	4.2331	0.07987	53	583.93	1.374	0.045436	
TRT	1.3586	0.67928	2	5.96	111.6853	0.008668	**
LINE:TRT	5.6824	0.05361	106	581.74	0.9222	0.692286	

G. Leaf C18:0 fatty acid content

Source of Variation	Sum Sq	Mean Sq	NumDF	DenDF	F value	Pr(>F)	Sig.
LINE	6.5858	0.12426	53	584.25	1.9458	1.298e-4	***
TRT	0.5629	0.281453	2	5.99	4.4074	0.06656652	.
LINE:TRT	5.8031	0.054747	106	581.25	0.8573	0.835758	

H. Plant water content (PWC)

Source of Variation	Sum Sq	Mean Sq	NumDF	DenDF	F value	Pr(>F)	Sig.
LINE	0.73864	0.01394	53	591.23	2.7545	3.201e-9	***
TRT	1.02723	0.51362	2	5.97	101.5158	2.460e-5	***
LINE:TRT	0.99548	0.00939	106	589.61	1.8562	3.854e-6	***

I. Leaf carbon isotope $\Delta^{13}C$

Source of Variation	Sum Sq	Mean Sq	NumDF	DenDF	F value	Pr(>F)	Sig.
LINE	87.064	1.6427	53	584.18	4.1733	2.2e-16	***
TRT	30.148	15.0742	2	5.99	38.2956	3.861e-4	***
LINE:TRT	43.707	0.4123	106	583.02	1.0475	0.364401	

J. Total Antioxidant Capacity

Source of Variation	Sum Sq	Mean Sq	NumDF	DenDF	F value	Pr(>F)	Sig.
LINE	12.3589	0.23319	53	273.23	15.59	<0.0001	***
TRT	0.4515	0.22573	2	4.23	15.09	0.0119	*
LINE:TRT	1.4989	0.01414	106	264.22	0.95	0.6258	

K. Leaf nitrogen $\Delta^{15}N$

Source of Variation	Sum Sq	Mean Sq	NumDF	DenDF	F value	Pr(>F)	Sig.
LINE	45.969	0.86734	53	585.39	1.2253	0.1389	
TRT	2.272	1.1359	2	6	1.6047	0.2766	
LINE:TRT	88.651	0.83633	106	583.88	1.1815	0.1206	

Supplementary Table S3. Statistical analysis of seed fatty acid composition for the camelina diversity panel grown in multi-location field trials. F-values are approximate resulting from a LMM with denominator degrees of freedom (ddf) estimated using the Kenward-Roger approximation. Variables correspond to fatty acid molecular species (number of carbons: desaturations). UK, United Kingdom; FR, France; ITA, Italy.

Variable	Country	ddf	F-values	P-value
C16:0	UK	147.89	4.053	<0.0001
C18:0	UK	167.19	8.2307	<0.0001
C18:1	UK	167.19	5.7279	<0.0001
C18:2	UK	167.19	15.974	<0.0001
C18:3	UK	167.16	7.2979	<0.0001
C20:0	UK	167.19	19.67	<0.0001
C20:1	UK	167.07	4.0634	<0.0001
C20:2	UK	167.09	36.536	<0.0001
C20:3	UK	167.12	15.411	<0.0001
C22:1	UK	167.11	18.728	<0.0001
Others	UK	148.01	5.6294	<0.0001
C16:0	FR	158.01	16.351	<0.0001
C18:0	FR	158.01	18.369	<0.0001
C18:1	FR	158.01	18.235	<0.0001
C18:2	FR	158.01	14.514	<0.0001
C18:3	FR	158	21.087	<0.0001
C20:0	FR	158	12.282	<0.0001
C20:1	FR	158.01	19.692	<0.0001
C20:2	FR	158	41.691	<0.0001
C20:3	FR	158.01	12.736	<0.0001
C22:1	FR	158	30.472	<0.0001
Others	FR	158	10.076	<0.0001
C16:0	ITA	125.17	9.7513	<0.0001
C18:0	ITA	125.2	17.42	<0.0001
C18:1	ITA	125.18	18.032	<0.0001
C18:2	ITA	125.18	10.228	<0.0001
C18:3	ITA	125.18	16.606	<0.0001
C20:0	ITA	125.2	15.172	<0.0001
C20:1	ITA	125.18	12.116	<0.0001
C20:2	ITA	128.18	19.981	<0.0001
C20:3	ITA	125.2	9.1827	<0.0001
C22:1	ITA	125.19	21.81	<0.0001
Others	ITA	125.18	10.356	<0.0001

Supplementary Table S4. Evaluation of selected camelina lines in field trials with contrasting responses to early-stage abiotic stress. (A) Drought, and (B) heat stress. Lines were selected based on their distribution in the global PCA 2D space and colour coded based on cos2 values (Fig. 5 – red and orange; outliers, and blue; central). For each line details of the metabolic marker response to abiotic stress are provided. Lines were ranked low to high, where 1 is the largest number of responsive metabolic markers, and 54 is the smallest or no response (combined accumulated or depleted markers when compared to ambient conditions, Fig. 3). In addition, agronomic traits determined in multiple location field trials (Italy, France and UK; Supplementary Fig. S6) are provided for the best and worst performing camelina lines at each location (maximum and minimum values for yield, TGW and seed oil content; values highlighted in red and green indicate poor/good respectively in comparison to max/min values)

Supplementary Table S4A. Evaluation of selected camelina lines in field trials with contrasting responses to early-stage drought stress. Green and purple shading show highest and lowest values, respectively, across three countries.

Camelina Panel Candidate Selection	(1) Early-stage Abiotic Stress Drought v Ambient			(2) Agronomic Traits in Multi-location Field Trials								
	Global PCA Coordinates		Stress Responsive Metabolic Markers	Yield (g/m^2)			TGW (g)			Oil content (%)		
UNT Line	PC1	PC2	Ranking (1 to 54)	ITA	FR	UK	ITA	FR	UK	ITA	FR	UK
22	7.36	4.14	14	315.3	82.6	153.3	0.955	0.801	0.796	38.3	40.4	39.0
5	5.95	0.73	36	275.2	109.0	151.0	1.324	1.128	0.956	39.9	38.6	37.2
59	5.61	-1.29	4	470.3	83.3	167.9	0.901	0.738	0.749	38.6	39.3	38.7
31	2.97	7.85	46	247.4	84.6	122.6	1.005	0.799	0.800	39.4	38.2	36.9
35	-2.80	-6.88	40	320.4	74.4	114.1	1.123	0.938	0.929	37.2	39.0	37.9
28	-4.52	1.57	23	162.2	151.3	180.8	1.112	1.153	1.119	38.1	42.0	42.7
18	-4.06	5.86	27	313.9	91.4	147.3	1.229	1.114	0.946	38.0	42.2	38.3
1	0.75	-0.32	29	419.1	103.9	153.1	1.058	0.820	0.789	39.2	40.4	38.8
34	0.60	-0.30	30	298.6	108.0	152.3	1.237	1.046	0.997	40.0	40.0	37.7
13	-0.01	-0.94	31	332.6	101.5	172.6	1.253	0.974	0.926	39.3	39.1	37.6
52	-0.21	-0.41	21	251.0	108.0	171.8	0.998	0.918	0.880	36.5	39.4	37.8
50	-0.48	0.32	17	310.5	98.9	143.4	1.075	0.835	0.869	37.5	39.3	39.4
8	-1.61	0.19	19	323.3	110.6	128.5	1.436	1.232	1.156	40.5	43.1	40.8
Camelina Panel Line with Maximum Value				40	44	29	57	57	57	53	8	28
Camelina Panel Maximum Value				599.4	155.2	191.6	1.556	1.299	1.269	41.2	43.1	42.7
Camelina Panel Line with Minimum Value				3	7	56	33	21	59	26	10	47
Camelina Panel Minimum Value				27.3	40.8	86.4	0.901	0.737	0.749	33.9	37.0	34.9

Supplementary Table S4B. Evaluation of selected camelina lines in field trials with contrasting responses to early-stage heat stress. Green and purple shading show highest and lowest values, respectively, across three countries.

Camelina Panel Candidate Selection	(1) Early-Stage Abiotic Stress – Heat v Ambient			(2) Agronomic Traits in Multi-Location Field Trials								
	Global PCA Coordinates		Stress Responsive Metabolic Markers	Yield (g/m^2)			TGW (g)			Oil Content (%)		
UNT Line	PC1	PC2	Ranking (1 to 54)	ITA	FR	UK	ITA	FR	UK	ITA	FR	UK
31	7.65	10.37	37	247.4	84.6	122.6	1.005	0.799	0.800	39.4	38.2	36.9
18	-2.96	3.24	6	313.9	91.4	147.3	1.229	1.114	0.946	38.0	42.2	38.3
50	-4.17	0.56	28	310.5	98.9	143.4	1.075	0.835	0.869	37.5	39.3	39.4
56	6.69	5.64	50	236.9	72.6	86.4	1.256	0.993	0.934	39.9	40.8	38.1
9	0.84	0.28	13	289.5	83.7	161.0	1.229	1.130	1.016	40.9	42.3	39.6
3	0.37	-0.34	4	27.3	100.1	154.5	1.185	0.979	0.997	35.7	39.9	38.4
38	-0.35	1.26	5	128.0	84.7	134.5	1.398	1.059	1.019	37.8	40.2	40.3
13	-0.56	0.87	40	332.6	101.5	172.6	1.253	0.974	0.926	39.3	39.1	37.6
8	-0.67	0.86	19	323.3	110.6	128.5	1.436	1.232	1.156	40.5	43.1	40.8
52	-0.87	-1.19	48	251.0	108.0	171.8	0.998	0.918	0.880	36.5	39.4	37.8
59	-1.36	0.83	3	470.3	83.3	167.9	0.901	0.738	0.749	38.6	39.3	38.7
	Camelina Panel Line with Maximum Value			40	44	29	57	57	57	53	8	28
	Camelina Panel Maximum Value			599.4	155.12	191.6	1.556	1.299	1.269	41.2	43.1	42.7
	Camelina Panel Line with Minimum Value			3	7	56	33	21	59	26	10	47
	Camelina Panel Minimum Value			27.3	40.8	86.4	0.901	0.737	0.749	33.9	37.0	34.9

Supplementary Table S5. Statistical analysis of agronomic parameters measured for the camelina diversity panel grown in multi-location field trials. F-values are approximate F-values resulting from a linear mixed model (LMM) with denominator degrees of freedom (ddf) estimated using the Kenward-Roger approximation (TGW, Thousand Grain Weight; Oil, oil content % of seed weight; FA, Fatty Acid; PUFA, Polyunsaturated Fatty Acid).

Variable	Country	ddf	F-values	P-value
Yield	UK	167.02	4.3887	<0.0001
Density	UK	167.05	1.5454	0.01992
Height	UK	167.06	2.9847	<0.0001
TGW	UK	167.04	13.193	<0.0001
Oil	UK	167.19	6.0372	<0.0001
Total FA	UK	167.16	1.9303	0.0009
Relative PUFA	UK	167.19	8.8808	<0.0001
Yield	FR	158	2.1426	0.0002
Density	FR	159	1.3303	0.09087
Height	FR	159	1.9739	0.0007
TGW	FR	158	39.516	<0.0001
Oil	FR	158	8.6888	<0.0001
Total FA	FR	158	1.383	0.06489
Relative PUFA	FR	158.01	21.664	<0.0001
Yield	ITA	125.19	5.4988	<0.0001
Density	ITA	125.19	9.045	<0.0001
Height	ITA	126.09	5.3536	<0.0001
TGW	ITA	124.3	13.7	<0.0001
Oil	ITA	125.19	7.1571	<0.0001
Total FA	ITA	125.17	2.6609	<0.0001
Relative PUFA	ITA	125.2	14.542	<0.0001

Supplementary Table S6. Coefficients of variation (CV; percentage) for parameters measured in the camelina diversity panel. (A) for individual metabolites in early-stage abiotic stress experiments and (B) agronomic performance parameters recorded in multi-location field trials.

A. Calculated CV values (%) for metabolites in the early-stage abiotic stress glasshouse experiments

Metabolites	Ambient	Drought	Heat
Amino Acids	24.2	24.6	27.2
Citrate	44.7	51.2	45.1
Glucose	91.9	92.9	70.5
Malate	21.0	17.7	19.8
Protein	23.9	21.6	20.5
Starch	63.1	82.3	46.2
Sucrose	30.0	27.0	24.4
Chlorophyll *a*	31.7	34.2	34.0
Chlorophyll *b*	72.7	58.4	34.4
Polyphenols	24.4	25.0	23.7

B. Calculated CV values (%) for agronomic performance parameters (APP) in multi-location field trials

APP	France	Italy	UK
TGW (g)	13.9	12.5	13.8
Yield (g/m^2)	37.4	57.2	22.4
Oil Content (%)	4.4	5.1	5.1

Supplementary Table S7. Summary of the main dates and meteorological data at the three field trial locations. GDD, Growing Degree Days; minimum (T_{min}), mean (T_{mean}) and maximum (T_{max}) temperatures shown for each growing season. Green shading indicates the highest value for each parameter.

Location	Sowing date	Harvest date	From Sowing to Harvest						From 50% Flowering to Harvest					
			Cycle length (days)	GDD	Rainfall (mm)	T_{min} (°C)	T_{mean} (°C)	T_{max} (°C)	Cycle length (days)	GDD	Rainfall (mm)	T_{min} (°C)	T_{mean} (°C)	T_{max} (°C)
Bologna (IT)	29th October 2020	17th June 2021	231	1412	290.4	4.58	9.77	15.20	65	828	51.8	10.16	16.74	23.03
Harpenden (UK)	19th April 2021	11th August 2021	114	1155	239.8	9.64	14.13	18.62	57	721	137.2	12.54	16.65	20.75
Versailles (FR)	26th March 2021	23rd August 2021	150	1647	356.5	9.44	14.77	20.48	86	1253	224.0	13.26	18.34	23.87

Supplementary Methods

Diversity panel population structure analysis

SNPs with a minor allele frequency (MAF) <0.05 were discarded. After initial filtering, the genotype dataset comprised 353,552 biallelic SNPs for 230 camelina accessions (54 from this study and 176 public accessions). Prior to full analysis of the population structure the data set was further filtered to retain only SNPs that were bi-allelic, had a read quality score of at least 20, a minor allele frequency of at least 0.05, and at least three sequencing reads aligned to the respective genomic position. In addition, further filtering retained genomic positions or sites that had an acceptable rate of heterozygosity i.e., ≤50%. Finally, sites correlated due to linkage disequilibrium were also removed. To complete the population structure analysis PCA was applied to the genotypic dataset (sikit-allel; Miles et al. 2024). The resulting set of SNPs were subjected to noise reduction and then k-means clustering algorithm (sklearn package; Pedregosa et al. 2011), visualised using plotly (Inc., P. T. 2015). Pairwise genetic distances were calculated for all pairs of accessions using Identity by State (IBS) distance measures (PLINK program version 1.9 (Purcell et al. 2007) and these IBS-distances (plink parameter “1-IBS”) were used to carry out hierarchical clustering (Hartigan 1975) in R (hclust). To infer the best performing number of subpopulations we examined the cross-validation error (CVE) reported by ADMIXTURE, choosing the parameter value with the lowest CVE. To visualize the results of ADMIXTURE barplots were produced in which each accession was represented by a single bar sub-divided into coloured sections. Each section represents the fraction of a genome coming from ancestry belonging to the subpopulation represented by that colour. The barplots were then aligned with the dendrogram generated by the hierarchical clustering analysis (based on IBS distances), plotted in R (version 4.2.3; R Core Team 2023). Finally, we carried out a PCA using the top 20 PCs of the variance-standardized relationship matrix (plink; v1.9). For each accession we obtained its respective coordinates in the PC vector space, of which the first two were used for scatterplots. Subsequently, k-means clustering was carried out on the accession coordinates in the 20-dimensional PC space (Hartigan and Wong, 1979). The best performing value was inferred by visual inspection of the Silhouette plot (Lengyel and Botta-Dukát, 2019). k-means clustering was done in R (version 4.2.3; R Core Team 2023).

Early-stage drought and heat experiments

Plants were germinated with supplemental lighting and 26°C to increase efficiency, eight days after sowing (DAS) seedlings were transplanted to individual pre-watered pots. All

plants were grown under well-watered and ambient temperature until 18 DAS, when the stress period started by increasing temperature in heat stress compartments and water-withdrawal in the drought stress pots (Supplementary Fig. S1). SWC was calculated as (FW-DW)/(SW-DW)x100, fresh weight (FW) was measured every day, saturated weight (SW) was measured by saturating each pot with water, and dry weight (DW) after drying the soil in each pot at 105°C until constant weight. For replication, four to five plants per line and treatment were grown, a total of 810 plants were distributed through six greenhouse compartments using a randomised design with sub-blocking structure Greenhouse/Bench/Tray/Pot. To ensure a small collection window and minimal impact on downstream analysis, collection was performed over a period of three days (days seven to ten after stress was imposed) with 90 plants per treatment collected each day (270 plants per day; 1/3 of the plants in each treatment collected in each of the three collection days). One (ambient and heat stress) or two (drought stress) leaves per plant were collected for antioxidant and metabolomic analysis, and one leaf disc per plant collected for fatty acid analysis. Leaf tissue was immediately frozen using liquid nitrogen and kept in -80°C until analysis. The remaining tissue was collected above the cotyledons, fresh weight registered and transferred to an oven at 60°C to assess plant biomass and further carbon isotope analysis. Two independent experiments were performed.

Multi-location agronomic field trials

In Italy, the trial was conducted at the experimental farm of Bologna University set in Cadriano (44° 33’ N, 11° 23’ E, 33 m a.s.l.), which is characterized by a cumulative annual precipitation of 613 mm and a long-term mean annual temperature of 13.2°C. The soil of the trial was silty-clay-loam (29% sand, 45% clay, 26% silt, pH = 8.07, soil organic matter = 1.82% DM). N fertilization (urea) was manually supplied at the beginning of stem elongation, at a rate of 50 kg/ha. The trial was rainfed, no herbicides nor pesticides were necessary. At full crop maturity, plants were manually cut at soil level and then threshed using a mechanical plot combine to separate straw and seed. Representative straw and seed samples were collected from each plot and dried in an oven at 105°C to determine residual moisture.

In UK, the trial was conducted in the Sawyers 2 field at the Rothamsted Farm in Harpenden (51° 49’ 0.27647”, -0° 22’ 29.03827”, 132 m a.s.l.), characterised by a cumulative annual precipitation of 702.5 mm and a mean annual temperature of 10.3°C in 2021. The soil type was clay loam, and the preceding crop was spring wheat. The field trial location was pre-treated with herbicides (four weeks before drilling), and no herbicides or pesticides were applied once the trial started. Nitrogen (N, 100 kg/ha), sulphur (S, 12 kg/ha), phosphorus (P,

40 kg/ha), and potassium (K, 50 kg/ha) were all applied just after drilling. The trial area was covered with a fleece for the first three weeks to aid establishment. Plants were harvested on 11/08/2021 at full crop maturity, 114 days after sowing, using a Haldrup C-65 small plot combine harvester. Representative straw and seed samples were collected from each plot and put in an oven at 80°C to determine residual moisture.

In France, the trial was conducted at the INRAE experimental farm (48.806341° N, 2.085599° E, 115.93 m a.s.l.), which is characterized by a cumulative annual precipitation of 772 mm and a long-term mean annual temperature of 11.1°C. The soil of the trial was silty-clay-loam (20% sand, 19% clay, 61% silt, pH = 7.72, soil organic matter = 2.29% DM). Background fertiliser was supplied on 15th March 2021 with 200 kg/ha of 0/25/25/6 N/P/K/S, and nitrogen fertilizer was supplied before sowing at a rate of 150 kg/ha with ammonium nitrate 33.5% (50 units N). The trial was rainfed and no herbicide or pesticide were provided. At full maturity of the crop, plants were manually cut with a STIHL HL 94 CE telescopic hedge trimmer (length 60 cm) and then threshed using a mechanical thresher to separate straw and seed.

Fatty Acid Analysis

Protocol for FA analysis at INRAe AgroParisTech, France. A total of 20 seeds were weighed in a glass tube and mixed with 1.345 ml of fatty acid methyl ester (FAME) buffer [methanol (1 mL), toluene (0.3 mL), sulfuric acid (25 µL) and C17:0 internal standard (20 µL of 10 mg/mL solution)]. The tubes were closed and warmed at 90°C for 90 min. After cooling, water (1.5 mL) and hexane (0.5 mL) were added, and the mix was centrifuged for 5 min at 1600 g. 50 µL of the organic phase was diluted 1/20th in hexane (1 mL) and 50 µL was transferred into a GC vial for analysis by Gas Chromatography followed by Mass Spectrometry (GC/MS). The samples were injected on an Agilent 7890A gas chromatograph coupled to an Agilent 5975C mass spectrometer. The polar column was a BPX70 (SGE) (30 m). Oven temperature ramp was 16°C/min from 70°C to 160°C, then 4°C/min up to 240°C and 10°C/min up to 260°C for 4 min (run length 31,6 min). Helium constant flow was 1.52 mL/min. Temperatures were the following: injector 250 °C, transfer line 290 °C, source 250 °C and quadrupole 150 °C.

Protocol for FA analysis at RRes, UK. For the FAME analysis a two-step direct methylation protocol was used. Samples of three replicates per plot with 20 seeds each were weighed and placed in glass vials mixed with 2.0 mL of 1 mM/L of heptadecanoic acid (internal standard C17:0, Sigma-Aldrich®) methylation mix. The methylation mix was composed of 66% methanol, 28% toluene, 4% 2,2-Dimethoxypropane and 2% sulfuric acid. The capped vials were heated to 85°C for 4h, allowed to cool to room temperature, and 1 mL of 1%

sodium chloride was added. The samples were mixed, and lipids extracted into 0.5 mL 100% hexane. Following methylation, the hexane fraction was dried under continuous N_2 flow and re-suspended in 1 mL of hexane. FAMEs were analysed by Gas Chromatography (GC) (Agilent 7890A, Agilent Technologies) using an Agilent J&W 122-2332 column (30 m × 250 µm × 0.25 µm, Agilent Technologies) and detected using a Flame Ionisation Detector (FID). Inlet and detector temperature were set to 250°C and 1 µL of each sample was analysed using 15:1 split ratio injection and a constant flow rate of 1.5 mL/minute. The oven temperature cycle as start temperature of 150°C held for 2 minutes to allow vaporised samples and the solvent (hexane) to condensate at the front of the column. Oven temperature was then increased to 240°C at 10°C/minute. The final temperature of 240°C was held for one minute and 50s giving a total run time of 12 min and 50s per sample. Chromatograms were analysed using the offline session of the Agilent ChemStation software (Agilent Technologies, USA). The peak area of each FAME was normalized to the internal standard and further normalized to the weight of the initial sample. The retention time and identity of each peak was calibrated using the Supelco® 37 Component FAME Mix (certified reference material TraceCERT®, Sigma-Aldrich®). Total FA content was calculated as the sum of all FA present, relative FA composition as percentage of total, leaf relative polyunsaturated FA content (PUFA index) as [*PUFA* (C18:3, C18:2, C20:2)] / [*MUFA* (C18:1, C16:1, C20:1) + *SFA* (C18:0, C16:0, C20:0)]; MUFA, monounsaturated FA; SFA, saturated FA.

Metabolomic Analysis

Untargeted metabolic profiling by UHPLC-LTQ-Orbitrap mass spectrometry (LCMS) was performed as described previously (Dussarrat et al. 2021; Martins et al. 2022) using an Ultimate 3000 ultra-high-pressure liquid chromatography (UHPLC) system coupled to an LTQ-Orbitrap Elite mass spectrometer interfaced with an electrospray (ESI) ionisation source (ThermoScientific, Bremen, Germany). Reverse phase chromatographic separation, negative ionisation and MS settings were detailed previously (Martins et al. 2022). The LCMS analytical sequence included 950 sample runs (610 unique biological samples, 167 technical duplicates and 2 technical triplicates), 70 biological standards (prepared by mixing sample powders of camelina material to constitute a biological pool that is used for data interoperability) and 120 extraction blanks (prepared without plant material and used to rule out potential contaminants detected by untargeted metabolomics). All sample runs were randomised with a satisfactory level of biological replication (n = 4 or 5, except for one combination [line x condition] that was only duplicated). In addition, a Quality Ambient (QC) sample was prepared by mixing 20 µL from each sample and biological standard. QC

samples were injected every 12 samples (108 in total) and used for untargeted metabolomics for i) the correction of signal drift during long batches, and ii) the calculation of coefficients of variation for each metabolomic feature so only the most robust ones are retained for chemometrics (Broadhurst et al. 2018). Briefly, MS1 full scan acquisitions at high-resolution (240k) were performed on six QC samples for exact mass measurements, and all samples were subjected to MS2 Data Dependent Analysis (DDA, 30k resolution) to generate fragmentation information for further annotation (Martins et al. 2022). Raw LCMS data were processed using MS-DIAL v 4.8 (Tsugawa et al. 2015), yielding 11 680 RT-m/z features. After data-curation (blank check, SN > 10, CV QC < 30%), we retained 3016 final features for further chemometrics, of which 220 matched with MS1 and MS2 (Level 2 ID), 1446 suggested features matched with MS1 only (Level 3 ID), and 1350 unknowns (no match). MS-DIAL annotation of metabolic features was performed using the online library MSMS-Public-Neg-VS15.msp (36,848 records). Thus, putative annotation of differentially expressed metabolites resulted from MS-DIAL screening of the MS1 detected exact HR m/z and MS2 fragmentation patterns against multiple online databases (http://prime.psc.riken.jp/compms/msdial/main.html#MSP) (Tsugawa et al. 2015). InChiKeys of annotated features were used in ClassyFire to provide an automated structural ontology of chemical entities (Djoumbou Feunang et al. 2016). Retention time correction was also applied in MS-DIAL, and metabolomic signals were normalised by the QC samples injected every 12 samples. Missing values for targeted assays (0.5%) were estimated using k-nearest neighbours (KNN) based on similar samples - KNN (sample-wise). Prior to multivariate statistics, the curated dataset was normalised using MetaboAnalyst v 5.0 (Pang et al. 2021) by median normalisation, cube root transformation and Pareto scaling, resulting in a curated dataset for multivariate statistics of 950 samples x 3016 metabolomic features + 9 major compounds (extraction blanks, standards and QC samples were discarded for clarity of visualisation).

To assess the structural similarity between different layers of biological data, hierarchical clustering was performed separately on phylogenetic, agronomic, and metabolomic datasets (Fig.6). Metabolomic and agronomic dendrograms were constructed using Euclidean distance and Ward.D2 method as implemented in the pheatmap package in R. The phylogenetic dendrogram was derived from genomic data used in Fig. 1, pruned to keep only the genotypes for which both metabolomic and agronomic data were available. To compare the resulting dendrograms, Baker's Gamma correlation coefficient was calculated for each pair using the cor.dendlist function from the dendextend R (version 1.19.0) package (cite:10.1093/bioinformatics/btv428), with the method set to "baker". This coefficient quantifies the concordance in the relative ordering of sample pairs between two

dendrograms.
Metabolomic dendrograms were constructed independently for each environmental condition (control, drought stress, and heat stress) using hierarchical clustering based on Euclidean distances and Ward's method (ward.D2). The clustering was applied to metabolomic profiles collected under each specific condition. Agronomic parameters (seed yield (g/m²), thousand grain weight (TGW, g), and seed oil content (% of seed weight)) were averaged across all three field trial locations. These values were then mapped as color-coded annotations along the metabolomic dendrograms to visualize the relationship between metabolic clustering and field performance under each stress condition.

Stable Isotope Determination

The isotopic ratios were expressed as delta (□) units. For C, □□□□C ‰ = [(R_{sample}/$R_{standard}$)-1] x 1000, R_{sample} and $R_{standard}$ are the isotopic ratio 13C/12C of the sample and of the international Pee Dee Belemnite carbonate standard (PDB). For N, □□□N ‰ = [(R_{sample}/$R_{standard}$)-1] x 1000, R_{sample} and $R_{standard}$ are the isotopic ratio 15N/14N of the sample and of the international standard which is the N_2 atmospheric gas.

References:

Broadhurst, D., Goodacre, R., Reinke, S.N., Kuligowski, J., Wilson, I.D., Lewis, M.R., Dunn, W.B. Guidelines and considerations for the use of system suitability and quality control samples in mass spectrometry assays applied in untargeted clinical metabolomic studies. Metabolomics. 2018:**14:**72. https://doi.org/10.1007/s11306-018-1367-3

Hartigan, J.A. *Clustering Algorithms*. New York: Wiley. 1975.

Hartigan, J.A., Wong, M.A. Algorithm AS 136: A K-means clustering algorithm. Appl. Stat.. 1979:**28:**100–108. https://doi.org/10.2307/2346830

Lengyel, A., Botta-Dukát, Z. Silhouette width using generalized mean – A flexible method for assessing clustering efficiency. Ecol. Evo. 2019:**9**:13231–13243. https://doi.org/10.1002/ece3.5774

Martins, J., Pétriacq, P., Flandin, A., Gómez-Cadenas, A., Monteiro, P., Pinto, G., Canhoto, J. Genotype determines *Arbutus unedo* L. physiological and metabolic responses

to drought and recovery. Front. Plant Sci. 2022:**13**:1011542. https://doi.org/10.3389/fpls.2022.1011542

Miles, A., pyup.io bot, Rodrigues, M.F., Ralph, P., Kelleher, J., Schelker, M., Pisupati, R., Rae, S., Millar, T. cggh/scikit-allel: v1.3.8 (v1.3.8). Zenodo. 2024. https://doi.org/10.5281/zenodo.10876220

Pedregosa, F., Varoquaux, G., Gramfort, A., Michel, V., Thirion, B., Grisel, O., Blondel, M., Prettenhofer, P., Weiss, R., Dubourg, V., Vanderplas, J., Passos, A., Cournapeau, D., Brucher, M., Perrot, M., Duchesnay, E. Scikit-learn: Machine Learning in Python. J. Machine Learning Res. 2011:**12**(85):2825−2830. https://jmlr.csail.mit.edu/papers/v12/pedregosa11a.html

Tsugawa, H., Cajka, T., Kind, T., Ma, Y., Higgins, B., Ikeda, K., Kanazawa, M., VanderGheynst, J., Fiehn, O., Arita, M. MS-DIAL: data-independent MS/MS deconvolution for comprehensive metabolome analysis. Nat. Methods 2015:**12**:523–526. https://doi.org/10.1038/nmeth.3393
